# Novel Glycerophospholipid, Lipo- and *N*-acyl Amino Acids from Bacteroidetes: Isolation, Structure Elucidation and Bioactivity

**DOI:** 10.3390/molecules26175195

**Published:** 2021-08-27

**Authors:** Mona-Katharina Bill, Stephan Brinkmann, Markus Oberpaul, Maria A. Patras, Benedikt Leis, Michael Marner, Marc-Philippe Maitre, Peter E. Hammann, Andreas Vilcinskas, Sören M. M. Schuler, Till F. Schäberle

**Affiliations:** 1 Fraunhofer Institute for Molecular Biology and Applied Ecology (IME), Branch for Bioresources, 35392 Giessen, Germany; mona.bill@ime.fraunhofer.de (M.-K.B.); stephan.brinkmann@ime.fraunhofer.de (S.B.); markus.oberpaul@ime.fraunhofer.de (M.O.); maria.patras@ime.fraunhofer.de (M.A.P.); benedikt.leis@gmx.net (B.L.); michael.marner@ime.fraunhofer.de (M.M.); andreas.vilcinskas@ime.fraunhofer.de (A.V.); 2 Sanofi Pasteur, R&D, 69280 Marcy L’Etoile, France; MarcPhilippe.Maitre@sanofi.com; 3 Sanofi-Aventis Deutschland GmbH, R&D, 65926 Frankfurt am Main, Germany; peter.hammann@npconsult.me; 4 Evotec International GmbH, 37079 Göttingen, Germany; 5 Institute for Insect Biotechnology, Justus-Liebig-University of Giessen, 35392 Giessen, Germany

**Keywords:** linear lipoamino acid, lipid 430, lipid 654, *N*-acyl amino acid, lysophosphatidylethanolamine, bacteroidetes, *Chitinophaga*, *Olivibacter*, LC-MS/MS, antimicrobial lipids

## Abstract

The ‘core’ metabolome of the Bacteroidetes genus *Chitinophaga* was recently discovered to consist of only seven metabolites. A structural relationship in terms of shared lipid moieties among four of them was postulated. Here, structure elucidation and characterization via ultra-high resolution mass spectrometry (UHR-MS) and nuclear magnetic resonance (NMR) spectroscopy of those four lipids (two lipoamino acids (LAAs), two lysophosphatidylethanolamines (LPEs)), as well as several other undescribed LAAs and *N*-acyl amino acids (NAAAs), identified during isolation were carried out. The LAAs represent closely related analogs of the literature-known LAAs, such as the glycine-serine dipeptide lipids 430 (**2**) and 654. Most of the here characterized LAAs (**1**, **5**–**11**) are members of a so far undescribed glycine-serine-ornithine tripeptide lipid family. Moreover, this study reports three novel NAAAs (*N*-(5-methyl)hexanoyl tyrosine (**14**) and *N*-(7-methyl)octanoyl tyrosine (**15**) or phenylalanine (**16**)) from *Olivibacter* sp. FHG000416, another Bacteroidetes strain initially selected as best in-house producer for isolation of lipid 430. Antimicrobial profiling revealed most isolated LAAs (**1**–**3**) and the two LPE ‘core’ metabolites (**12**, **13**) active against the Gram-negative pathogen *M. catarrhalis* ATCC 25238 and the Gram-positive bacterium *M. luteus* DSM 20030. For LAA **1**, additional growth inhibition activity against *B. subtilis* DSM 10 was observed.

## 1. Introduction

Lipids are a diverse group of natural biomolecules. Thousands of distinct lipids, such as glycerolipids, sterol lipids, sphingolipids, lipoamino acids (LAAs), and phospholipids, are ubiquitous in all organisms. Each of them is chemically unique, and they exhibit different biological functions. Given the diversity in both the chemical and physical properties of lipids and the fact that each lipid type is involved at various stages of cellular processes, the definition of lipid function besides their primary biological role, i.e., the formation of cell membrane matrixes, is challenging. Described functions in cellular signaling, energy storage, or an implication as substrate for metabolite or protein lipidation are only examples [1].

Bacterial membrane composition differs among bacterial species and depends on the exposed environmental conditions [2]. In most cases and conditions studied, amphiphilic lipids such as glycerophospholipids are composed of two fatty acids, a glycerol moiety, a phosphate group, and variable head groups. Common examples are phosphatidylethanolamine (PE), phosphatidylglycerol (PG), cardiolipin (CL), lysyl-phosphatidylglycerol (LPG), phosphatidylinositol (PI), phosphatidic acid (PA), and phosphatidylserine (PS). Additionally, a small fraction of the bacterial membrane consists of lysophospholipids (LPLs). They are metabolic intermediates of bacterial phospholipid synthesis or they derive from membrane degradation. The most abundant member of this class is lysophosphatidylethanolamine (LPE). LPLs result from partial hydrolysis of phospholipids mediated by phospholipase A [3]. Other phosphorus-free lipids, such as sulfolipids, LAAs [2,4,5,6,7,8,9,10,11,12,13,14], or the growing lipid class of the *N*-acyl amino acids (NAAAs), are also reported [15]. The latter are found in all biological systems, but their functions remain unclear. It is hypothesized that these lipids are putative signaling molecules with a wide range of biological activities [16,17,18,19,20,21,22,23].

In general, a variety of physicochemical properties and bioactivities have been associated with all kinds of bacterial lipids, including hemagglutination [9], macrophage activation [10], bacterial virulence factors [24], involvement in the development of multiple sclerosis [25], and antimicrobial activities [26,27]. Moreover, simple representatives of the LAAs such as the glycine-serine dipeptide lipid 654 (also referred to as flavolipin [28], topostin D654 [29], and WB-3559 D [6,7]) and glycine-serine dipeptide lipid 430 were isolated from pathogenic Bacteroidetes strains associated with chronic periodontitis [30]. They account for osteoclast formation from RAW cells [31] and TLR2-dependent inhibition of osteoblast differentiation and function [32], and they are implicated in dendritic cell release of IL-6 mediated through engagement of TLR2 [33,34]. Furthermore, antimicrobial activity was reported for lipid 430 [35], the de-esterified enzymatic hydrolysis product of lipid 654 hydrolyzed by phospholipase A2 (PLA2) [36].

For enabling doubtless structure elucidation, various analytical methods such as MS and NMR are well established and intensively used in the field of natural product research in general. For lipids as one important sub-class, first, gas or liquid chromatography coupled to diverse mass spectrometry methods represent two key analytical techniques [37,38,39]. Second, NMR spectroscopy is not only widely utilized for structure elucidations of single lipids, successful applications regarding qualitative and quantitative analysis of lipids in complex mixtures are also reported in this context [40,41,42,43,44,45].

In a previous study, we identified the ‘core’ metabolome of the Bacteroidetes genus *Chitinophaga*, consisting of only seven metabolites [46]. We postulated a structural relationship among four of them based on their MS/MS fragmentation pattern, which suggested them to be unknown LAAs and LPEs. Here, we report the identification in total of 16 diverse lipids (11 LAAs, 2 LPEs, 3 NAAAs) produced by *Chitinophaga* spp. and *Olivibacter* sp. FHG000416, the ‘core’ ones included. Isolation and structure elucidation of nine of those lipids isolated from *Chitinophaga eiseniae* DSM 22224 [47] and FHG000416 was successfully achieved. Based on the four *Chitinophaga* core lipids, the known lipid 430, several novel LAA analogs thereof, and three undescribed NAAAs were characterized. In this context, we identified a novel glycine-serine-ornithine LAA family that is closely related to the previously described glycine and glycine-serine LAA families that lipid 430 and 654 belong to. Investigating them for their antimicrobial activity revealed a common growth inhibition effect against the Gram-negative pathogen *M. catarrhalis* ATCC 25238 and the Gram-positive bacterium *M. luteus* DSM 20030, with LAA **1** as the most potent one, also showing growth-inhibiting activity against *B. subtilis* DSM 10.

## 2. Results

### 2.1. Lipoamino Acids

A previous study revealed a small core metabolome of the Bacteroidetes genus *Chitinophaga*, identified as a genus of underexplored talented producers of natural products. The analyzed 25 strains commonly share only 7 of the 2736 identified metabolite buckets. Four of those seven ‘core’ metabolites correspond to the UHR-ESI-MS ion peaks at *m/z* 545.3908 [M + H]^+^, 452.2769 [M + H]^+^, 440.2767 [M + H]^+^, and 767.5896 [M + H]^+^, with retention times (RTs) of 11.2, 11.7, 12.1, and 17.4 min, respectively (Table S1). Based on their MS/MS fragmentation pattern, similar lipid moieties were postulated, suggesting a structural relationship [46].

**Figure 1:**
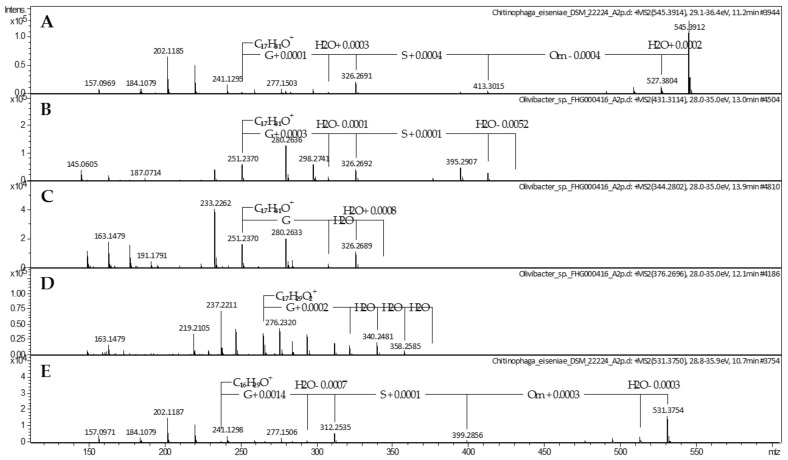
MS/MS spectra of lipoamino acids **1**–**4** (**A**–**D**) and **7** (**E**) produced by *Chitinophaga eiseniae* DSM 22224 (**1**, **7**) and *Olivibacter* sp. FHG000416 (**2**–**4**).

**Figure 2:**
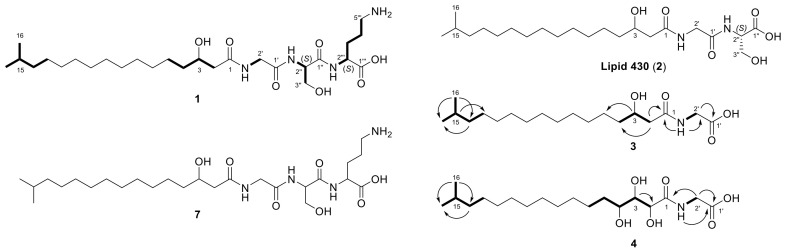
Chemical structures of lipoamino acids **1**–**4** and **7** produced by *C. eiseniae* DSM 22224 (**1**, **7**) and *Olivibacter* sp. FHG000416 (**2**–**4**). The COSY (in bold) and key H→C HMBC (arrows) correlations observed for lipids **1**, **3**, and **4** are indicated.

For the first core metabolite, the molecular formula C_27_H_52_N_4_O_7_ (**1**) was assigned according to the UHR-ESI-MS ion peak at *m/z* 545.3908 [M + H]^+^. The MS/MS fragmentation of **1** revealed neutral losses of H_2_O, followed by the three amino acids ornithine, serine, and glycine, resulting in the fragment ion of *m/z* 251.2366 [M + H]^+^. The corresponding ion formula of C_17_H_31_O^+^ indicated a fatty acyl group based on the molecular composition and apparent carbon-to-hydrogen ratio (Figure 1A). A missing hit in a database search indicated the potential novelty of LAA **1**, containing a glycine-serine-ornithine tripeptide. However, very close relatives, such as lipid 430 (**2**) and lipid 654, belong to the glycine-serine dipeptide LAA family, known to be biosynthesized by several bacteria, including representatives of the phylum Bacteroidetes [14,30,34]. Therefore, the metabolomics data generated in our previous study was examined for the presence of both dipeptide lipids. A corresponding ion peak for lipid 430 (**2**) at *m/z* 431.3114 [M + H]^+^ with a RT of 13.0 min was identified in 23 of the analyzed 25 *Chitinophaga* metabolomes (Table S1). Similar serial neutral losses of H_2_O, serine, and glycine, together with the same remaining fragment ion of *m/z* 251.2367 [M + H]^+^, strongly suggested **1** being a close derivative of **2**, expanded by an additional ornithine residue (Figure 1B). To provide a sufficient amount of the compounds for structure confirmation via NMR, our in-house Bacteroidetes-based extract library was examined for enhanced production of **1** and **2**. Lipid 430 (**2**) was enriched in extracts of strain FHG000416, which was assigned by 16*S* rRNA sequence analysis to the Bacteroidetes genus *Olivibacter* due to a sequence identity of ~94.5% towards *Olivibacter domesticus* DSM 18733 [48,49]. Extracts from both strains, *C. eiseniae* DSM 22224 and FHG000416, were considered as starting points for compound isolation. NMR analysis confirmed compound **2** to be lipid 430 since the data were in good agreement with the literature (Table 1) [35]. Compared thereto, ^1^H and 2D spectra of **1** were highly similar. Accordingly, the fatty acyl motif linked to glycine and serine was identified as *iso*-heptadecanoic acid (*iso*-C_17:0_). An additional amide proton 2′′′-NH (*δ*_H_ 7.66–7.59 ppm) was observed showing COSY correlation to methine proton H-2′′′ (*δ*_H_ 3.82–3.75 ppm, *δ*_C_ 53.6 ppm). Further correlations to methylene protons H-3′′′, H-4′′′, and H-5′′′ confirmed the presence of a *C*-terminal ornithine residue, as postulated based on the MS/MS fragmentation, identifying lipid **1** as 5-amino-2-(3-hydroxy-2-(2-(3-hydroxy-15-methylhexadecanamido)acetamido)propanamido)pentanoic acid (Figure 2; Table 1).

The stereochemistry of the chiral amino acids incorporated in LAA **1** and lipid 430 (**2**) was determined by advanced Marfey’s analysis, using *N*_α_-(2,4-dinitro-5-fluorophenyl)-l-valinamide (l-FDVA) as Marfey’s reagent [50]. d- and l-enantiomers of serine and ornithine served as RT references. In agreement with published data, the serine residue of lipid 430 (**2**) was identified as l-serine (Figure S1) [34]. This was also true for lipid **1**, which furthermore contains l-ornithine (Figure S1; Figure S2).

During processing, the extracts of FHG000416, two further derivatives of **1** and **2**, were recognized and identified based on their similar MS/MS fragmentation patterns. The two ions of *m/z* 344.2802 [M + H]^+^ and *m/z* 376.2696 [M + H]^+^ were assigned to putative lipids with molecular formulae C_19_H_37_NO_4_ (**3**) and C_19_H_37_NO_6_ (**4**). Compared with **1** and **2**, the MS/MS fragmentation of **3** showed the identical resulting fragment ion at *m/z* 251.2370 [M + H]^+^ after neutral losses of two water and one glycine molecule (Figure 1C). Therefore, we assumed that **1**, **2**, and **3** share the same acyl chain and glycine residue. In contrast, the MS/MS fragmentation pattern of **4** also showed admittedly the neutral loss of a glycine but also the loss of several water molecules. The resulting fragment ion at *m/z* 265.2162 [M + H]^+^ corresponded to the formula of C_17_H_29_O_2_^+^, indicating modifications in the acyl residue (Figure 1D). Finally, 1D and 2D-NMR spectra confirmed an *iso*-C_17:0_ aliphatic acyl group linked to glycine via a peptide bond (Table 1). In the case of lipid **3**, the α-methylene protons of the acyl group H-2 (*δ*_H_ 2.19 ppm) showed COSY correlation with the single methine proton H-3 (*δ*_H_ 3.79–3.74 ppm), suggesting substitution. The chemical shift of the HSQC-correlating carbon atom C-3 (*δ*_C_ 67.4 ppm) furthermore supported the hypothesis of a hydroxyl group being attached in β-position (Table 1). Thus, **3** was identified as (3-hydroxy-15-methylhexadecanoyl)glycine (Figure 2). For derivative **4**, NMR spectra undergirded the expected similarities, except for the methine proton H-3 (*δ*_H_ 3.44 ppm, *δ*_C_ 74.5 ppm) showing COSY correlations with methine instead of methylene protons in α- (H-2, *δ*_H_ 4.19 ppm) and *γ*-position (H-4, *δ*_H_ 3.40–3.35 ppm) of the acyl chain. In agreement with the chemical shift of the HSQC-correlating carbon atoms C-2 and C-4 (*δ*_C-2_ 70.9 ppm, *δ*_C-4_ 69.5 ppm), hydroxylation was determined at both sites, thereby identifying **4** as (2,3,4-trihydroxy-15-methylhexadecanoyl)glycine (Figure 2; Table S2).

**Figure 3:**
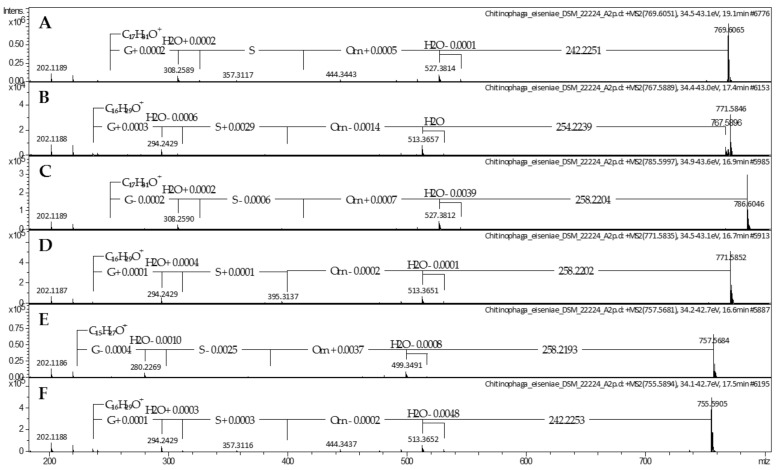
MS/MS spectra of lipoamino acids **5** (**A**), **6** (**B**), and **8**–**11** (**C**–**F**) produced by *Chitinophaga eiseniae* DSM 22224.

**Figure 4:**
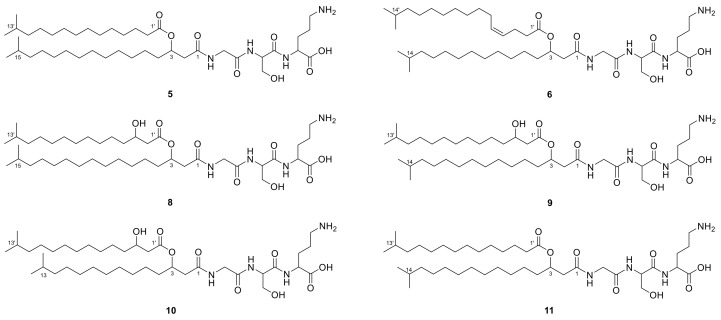
Postulated chemical structures based on UHR-ESI-MS/MS spectra of lipoamino acids **5**, **6**, and **8**–**11**, produced by *Chitinophaga eiseniae* DSM 22224.

In contrast with its de-esterified hydrolysis product lipid 430 (**2**) [36], lipid 654 (*m/z* 655.525 [M + H]^+^) carrying an additional ester-linked *iso*-pentadecenoic acid (*iso*-C_15:0_) was not observed in our previously generated *Chitinophaga* extracts (Table S1) [46]. Nevertheless, we searched for corresponding lipid 654 analogs because LAA **1** represents a close derivative of lipid 430 (**2**) containing an additional ornithine amino acid attached to the glycine-serine dipeptide. In 8 of 25 *Chitinophaga* metabolomes, the corresponding parent ion at *m/z* 769.6051 [M + H]^+^ with molecular formula C_42_H_80_N_4_O_8_ (**5**) was found (Table S1). Neutral losses of ornithine-serine-glycine and the resulting fragment ion at *m/z* 251.2372 [M + H]^+^ were identical to the MS spectrum of compound **1** (Figure 3A). Moreover, the MS/MS fragmentation pattern containing the characteristic fragment ion at *m/z* 527.3814 [M + H]^+^ (loss of 242.2251 Da (C_15_H_30_O_2_)) proved the presence of the expected ester-linked fatty acyl moiety (*iso*-C_15:0_) at C-3. Therefore, compound **5** (2-(3-aminopropyl)-5-(hydroxymethyl)-26-methyl-12-(12-methyltridecyl)-4,7,10,14-tetraoxo-13-oxa-3,6,9-triazaheptacosanoic acid) was clearly identified as the ornithine analog of the literature-known lipid 654 [34] (Figure 4).

Based on this finding, the second *Chitinophaga* core metabolite with a UHR-ESI-MS ion peak at *m/z* 767.5896 [M + H]^+^ was assigned the molecular formula C_42_H_78_N_4_O_8_ (**6**). Compared with compound **5**, a difference of 2.0155 Da indicated a double bond in one of the fatty acid residues. The MS/MS fragmentation pattern revealed the amino acid sequence ornithine-serine-glycine. Furthermore, compared with lipid **5**, a fragment ion at *m/z* 513.3657 [M + H]^+^ resulted from the loss of an ester-linked fatty acid residue (254.2239 Da) with additional 11.9988 Da (1× C) at C-3. Together with the remaining fragment ion at *m/z* 237.2212 ([M + H]^+^, C_16_H_29_O^+^, 14.0160 Da less compared with **1**–**3** and **5**), we postulate lipid **6** to carry two *iso*-palmitic acid (*iso*-C_16_) residues, a saturated amide-linked *iso*-C_16:0_, and an ester-linked mono-unsaturated *iso*-C_16:1_ (Figure 3B). In line with reported glycine and glycyl-serine analogs [13], we assume the double bond to be in Δ^4′^ position and with *Z* configuration for lipid **6**, identifying it as (*Z*)-2-(3-aminopropyl)-5-(hydroxymethyl)-27-methyl-12-(11-methyldodecyl)-4,7,10,14-tetraoxo-13-oxa-3,6,9-triazaoctacos-17-enoic acid (Figure 4). However, structural confirmation by NMR was not possible due to a low production titer in large-scale fermentation of *C. eiseniae* DSM 22224.

With lipid **6** carrying a different amide-linked fatty acid residue than **5**, we assumed to find a correlating close derivative of lipids **1**–**3** that carried an amide-linked *iso*-C_16:0_ fatty acid residue instead of an *iso*-C_17:0_. In total, 18 *Chitinophaga* strain data sets contained the matching positive parent ion at *m/z* 531.3754 according to molecular formula C_26_H_50_N_4_O_7_ (**7**) (Table S1). A missing ester-linked fatty acid residue and an otherwise identical UHR-ESI-MS/MS spectrum of compound **7** compared with lipid **6** confirmed the presence of an *iso*-C_16:0_ fatty acid residue amide linked to a glycine-serine-ornithine amino acid motif (Figure 1E). Therefore, lipid **7** was identified as 5-amino-2-(3-hydroxy-2-(2-(3-hydroxy-14-methylpentadecanamido)acetamido)propanamido)pentanoic acid (Figure 2).

In addition to lipids **5** and **6**, we observed four closely related metabolites with similar masses and RTs: **8** (*m/z* 785.6016 [M + H]^+^, C_42_H_80_N_4_O_9_, 16.9 min), **9** (*m/z* 771.5852 [M + H]^+^, C_40_H_78_N_4_O_9_, 16.7 min), **10** (*m/z* 757.5681 [M + H]^+^, C_40_H_76_N_4_O_9_, 16.6 min), and **11** (*m/z* 755.5894 [M + H]^+^, C_41_H_78_N_4_O_8_, 17.5 min) in the extract of *C. eiseniae* DSM 22224. Sharing the same tripeptide moiety of glycine-serine-ornithine, these lipid 654 analogs vary in the length and hydroxylation of their acyl chains. The analysis of the UHR-ESI-MS/MS spectra of LAAs **8**–**10** revealed a neutral loss of 258.2204 Da (C_15_H_28_O_3_). Compared with the neutral loss of 242.2251 Da (C_15_H_28_O_2_) of LAA **5**, the additional 15.9953 Da depicted the presence of an additional oxygen atom in the ester-linked *iso*-C_15:0_ fatty acid residue (Figure 3C–E). In accordance with the isolated LAAs **2** and **3**, the corresponding hydroxyl group was assigned to C-3′ position. With all other fragment ions identical to LAA **5**, the structure of LAA **8** was postulated as 2-(3-aminopropyl)-16-hydroxy-5-(hydroxymethyl)-26-methyl-12-(12-methyltridecyl)-4,7,10,14-tetraoxo-13-oxa-3,6,9-triazaheptacosanoic acid (Figure 4). LAA **9** shared the characteristic fragment ion at *m/z* 237.2213 [M + H]^+^, identified as an unsaturated amide-linked *iso*-C_16:0_ fatty acid residue with LAA **6** (Figure 3D). Therefore, **9** was identified as 2-(3-aminopropyl)-16-hydroxy-5-(hydroxymethyl)-26-methyl-12-(11-methyldodecyl)-4,7,10,14-tetraoxo-13-oxa-3,6,9-triazaheptacosanoic acid (Figure 4). For LAA **10**, a characteristic fragment ion was detected at *m/z* 223.2059 ([M + H]^+^, C_15_H_27_O^+^) with 14.0154 Da less compared with the one of **9** (Figure 3E). This indicated lipid **10** to be a LAA with an ornithine-serine-glycine tripeptide amide linked to an *iso*-C_15:0_ fatty acid residue that carries another C-3 ester-linked hydroxylated *iso*-C_15:0_ fatty acid residue. The chemical structure of the new compound **10** is 2-(3-aminopropyl)-16-hydroxy-5-(hydroxymethyl)-26-methyl-12-(10-methylundecyl)-4,7,10,14-tetraoxo-13-oxa-3,6,9-triazaheptacosanoic acid (Figure 4). The analysis of the UHR-ESI-MS/MS spectrum of the last derivative of this lipid family revealed the ester-linked fatty acid to have an *iso*-C_15:0_ and the amide-linked one an *iso*-C_16:0_ moiety without further modifications (Figure 3F). Thus, the new LAA **11** was identified as 2-(3-aminopropyl)-5-(hydroxymethyl)-26-methyl-12-(11-methyldodecyl)-4,7,10,14-tetraoxo-13-oxa-3,6,9-triazaheptacosanoic acid (Figure 4).

### 2.2. Phospholipids

**Figure 5:**
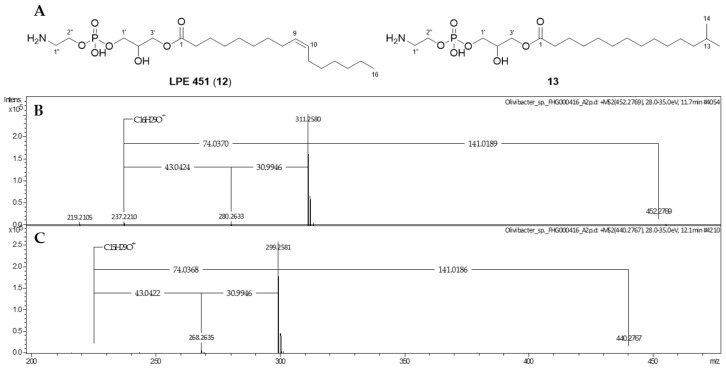
Chemical structures (**A**) and MS/MS spectra of LPE 451 (**12**, **B**) and its new derivative (**13**, **C**), isolated from *Olivibacter* sp. FHG000416.

In addition to the aforementioned LAAs, dereplication of the last two *Chitinophaga* core buckets with *m/z* 452.2769 [M + H]^+^ and 440.2767 [M + H]^+^ suggested the closely related molecular formulae C_21_H_42_NO_7_P (**12**) and C_20_H_42_NO_7_P (**13**), respectively. A database query for **12** provided LPE 451 as a structural hypothesis, based on similar MS and MS/MS spectra in accordance with the literature [51]. Neutral losses of the phosphatidylethanolamine group (141.018 Da) followed by the CH_2_OH group (30.994 Da) or the glycerol moiety (74.037 Da), which are reported for phospholipids such as the identified LPE [52,53], resulted in a key fragment ion at *m/z* 237.2210 [M + H]^+^ (C_16_H_29_O^+^, DBE = 3). Highly similar MS/MS fragmentation of **13** led to the fragment ion at *m/z* 225.2212 [M + H]^+^ (C_15_H_29_O^+^, DBE = 2), indicating the structural variance to be located in the acyl motif. For NMR analysis, the isolation of these compounds was again carried out from extracts of *Olivibacter* sp. FHG000416, as higher production titers were observed compared with *C. eiseniae* DSM 22224. For **12**, NMR data confirmed the occurrence of lysophosphatidylethanolamine (C_16:1_), as postulated by dereplication. According to published data for LPE 451, the double bond (*δ*_H-15/16_ 5.24–5.12 ppm) of the mono-unsaturated palmitoyl motif was assigned to Δ^9^ position with *Z* configuration, with the acyl chain being attached to the glycerol moiety at *sn*-1 position (Table S3) [51]. Thus, **12** was identified as 1-(9*Z*-palmitoyl)-2-hydroxy-*sn*-glycerol-3-phosphoethanol-amine (Figure 5). In comparison, 1D and 2D-NMR data acquired for **13** revealed the double bond of the acyl chain to be missing. Instead, an isopropyl moiety (*δ*_H-19_ 1.29 ppm, *δ*_C-20_ 27.7 ppm/*δ*_H-20_ 0.63 ppm, *δ*_C-20_ 22.2 ppm) was observed, identifying the acyl chain as *iso*-C_15:0_ attached to the otherwise identical molecule (Table S3). Therefore, **13** was identified as 1-isopentadecanoyl-2-hydroxy-*sn*-glycerol-3-phosphoethanolamine (Figure 5).

### 2.3. N-acyl Amino Acids from Olivibacter sp. FHG000416

**Figure 6:**
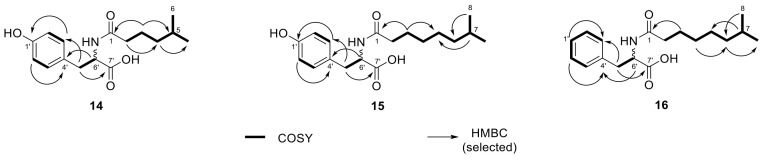
Chemical structures of *N*-acyl amino acids (**14**–**16**) isolated from *Olivibacter* sp. FHG000416, with observed COSY (in bold) and key H→C HMBC (arrows) correlations.

During processing extracts from *Olivibacter* sp. FHG000416, three additional metabolites with lipid-like properties (e.g., surface activity) were observed. For compound **14**, the molecular formula C_16_H_23_NO_4_ (*m/z* 294.1696 [M + H]^+^) was determined by UHR-ESI-MS. The MS/MS fragmentation pattern showed a fragment at *m/z* 182.0811, matching the protonated form of tyrosine ([M + H]^+^, C_9_H_12_NO_3_^+^). This hypothesis was further supported by neutral losses of NH_3_ and H_2_O, commonly known for the fragmentation of amino acids (Figure S3A) [15]. Furthermore, the neutral loss of 112.0885 Da (C_7_H_12_O) implied the loss of a saturated acyl group. The 1D and 2D NMR experiments confirmed this structural proposal (Table 2), showing an isopropyl group at the end of the *iso*-C_7:0_ acyl moiety, which was attached to tyrosine via a peptide bond. Therefore, **14** was identified as *N*-(5-methyl)hexanoyl tyrosine (Figure 6).

Based on UHR-ESI-MS analysis, compounds **15** (*m/z* 322.2013 [M + H]^+^, C_18_H_27_NO_4_) and **16** (*m/z* 306.2063 [M + H]^+^, C_18_H_27_NO_3_) shared the same saturated acyl group indicated by the neutral loss of 140.1201 Da (C_9_H_16_O). The difference of 28.0317 Da compared with **14** is equivalent to two additional methylene groups matching an *iso*-C_9:0_ acyl group. Identical to NAAA **14**, the acyl group of **15** was attached to tyrosine (Figure S3B). In contrast, the fragment ion of NAAA **16** at *m/z* 166.0862 ([M + H]^+^, C_9_H_12_NO_2_^+^) corresponded to one oxygen atom less and was thereby assumed to be phenylalanine (Figure S3C). NMR analysis confirmed both structural proposals based on the MS data. NAAA **15** was determined as *N*-(7-methyl)octanoyl tyrosine and **16** as *N*-(7-methyl)octanoyl phenylalanine (Figure 6, Table 2). Finally, advanced Marfey’s analysis of NAAAs (**14**–**16**) revealed that all three compounds had been isolated as an enantiomeric mixture. The d/l-ratio was determined by UV signal integration as 1:16 for **14**, 1:1.7 for **15**, and 1.6:1 for **16** (Figure S4–S6).

### 2.4. Antimicrobial Activity of Lipids Isolated from Bacteroidetes

The antimicrobial activity of compounds **1**–**4** and **12**–**16** isolated either from *C. eiseniae* DSM 22224 or *Olivibacter* sp. FHG000416 was determined by microbroth dilution assay against a panel of 12 indicator strains up to a test concentration of 64 µg/mL. No growth inhibition effect was observed for LAA **4** and the NAAAs **15** and **16**. *N*-(5-methyl)hexanoyl tyrosine (**14**) exhibited very low effect against Gram-negative *M. catarrhalis* ATCC 25238 and Gram-positive *M. luteus* DSM 20030 at the highest concentration of 64 µg/mL. In contrast, LAAs **1**–**3** and both LPEs (**12** and **13**) showed growth inhibiting activity in a range of 4–16 µg/mL and 16–64 µg/mL against *M. catarrhalis* ATCC 25238 and *M. luteus* DSM 20030, respectively. Moreover, LAA **1** inhibited *B. subtilis* DSM 10 up to 8 µg/mL and *C. albicans* FH2173 as well as *E. coli* ATCC 35218 up to 64 µg/mL—the latter only when tested in bicarbonate-supplemented screening medium (MHC) (Table 3).

## 3. Discussion

Bacterial small molecules are of great importance for medicinal, industrial, and agricultural applications [54]. Within this class of compounds, lipids represent a structurally diverse class of metabolites with a variety of biological functions [2,26,30]. Advances in LC-MS emerged the field of lipidomics, allowing high-throughput detection and sophisticated analysis of complex lipid samples. Identification of abundant known lipid classes and structure predictions of lipids new to science are possible. However, compared with NMR techniques, LC-MS measurements that require lower amounts of sample will not provide enough data to deduce the planar structure unambiguously [39].

In the current study, we described the characterization and structure elucidation of the unknown previously determined core lipids of the Bacteroidetes genus *Chitinophaga* [46]. Interestingly, we observed the highest production titers of three of the four lipids in extracts of *Olivibacter* sp. FHG000416, a strain belonging to another Bacteroidetes genus. Therefore, compound isolation was carried out from these two strains: *Olivibacter* sp. FHG000416 and *Chitinophaga eiseniae* DSM 22,224. Two of the four lipids are identified as LPEs, with **13** being an undescribed derivative of the literature-known LPE 451 (**12**). The remaining ones (**1** and **6**), together with several further derivatives thereof, are novel lipids classified as LAAs. The literature frequently described the production of LAAs by various bacteria, with some of them being isolated and chemically fully characterized [5]. These are often mono- or dipeptide lipids containing glycine, serine, ornithine, or glycine-serine as amino acid residues amide-linked to an *iso*-fatty acid ester at C-3 with different degrees of unsaturation [6,7,8,9,10,11,12]. Based on MS/MS and NMR experiments, the lipids **3** and **4** were identified as new LAA glycine derivatives. The *Chitinophaga* core lipids **1** and **6**, as well as lipids **5** and **8**–**11** form a LAA family with an undescribed tripeptide moiety of glycine-serine-ornithine. This novel tripeptide LAA family is most closely related to the intensively studied glycine-serine dipeptide lipids 654 and 430 (**2**). Interestingly, with lipid 430 (**2**), only the de-esterified hydrolysis product of lipid 654 [34] was detected in the methanolic extracts of 23 of 25 *Chitinophaga* strains generated in our previous study [46]. Recent intensive studies of human pathogenic Bacteroidetes such as *P. gingivalis* revealed both dipeptide lipids to engage TLR2 [34]. They are involved in the development of two chronic inflammatory diseases, including periodontitis and atherosclerosis [30]. Furthermore, studies implicated lipid 654 to be involved in the development of multiple sclerosis [25]. Therefore, further studies are necessary to investigate the effects of the here described novel tripeptide LAAs in terms of immune response and whether they might also be part of the cell membrane of pathogens such as *P. gingivalis*.

The same applies to the new *N*-acyl tyrosine and phenylalanine analogs. They belong to a growing family of microbial secondary metabolites isolated from bacteria [22,23], fungi [21], or from environmental DNA expressed in heterologous hosts such as *E. coli* [16,17,18,19,20]. It is hypothesized that these lipids are possible signaling molecules with a wide range of biological activities from anti-cancer therapy targets to antimicrobial lipids [16,17,18,19,20,21,22,23]. With no antimicrobial activity observed, further studies are necessary to elucidate their biological function.

In addition to the structure-associated as well as the target organism-oriented antimicrobial activities of lipids, a membrane destabilization mechanism has been investigated for several decades [26,55]. In this context, a ‘carpet’ mechanism is suggested, which results in a detergent-like membrane permeation and/or disintegration [55,56]. Based on the reported findings, we selected a panel of 12 microorganisms to cover a wide range of possible targets for the herein described NPs. The antimicrobial profiling of the herein tested lipids revealed a frequent growth inhibitory effect towards *M. catarrhalis*, with lipid **1** additionally showing growth inhibiting activity against *B. subtilis*. The more hydrophobic cell surface of *M. catarrhalis*, compared with the surfaces of, e.g., *E. coli* and *P. aeruginosa*, is believed to be the reason for high accessibility of hydrophobic agents to the cell surface [57,58,59]. Therefore, we assume *M. catarrhalis* to be the most susceptible pathogen towards a suggested membranolytic mode of action mediated by LAAs carrying a hydrophobic lipid moiety and a short hydrophilic amino acid moiety. The mode of action needs to be confirmed in further studies.

## 4. Materials and Methods

### 4.1. Isolation of Olivibacter sp. FHG000416

*Olivibacter* sp. FHG000416 is incorporated into the Fraunhofer strain collection. This strain was isolated in 2016 from termite carton nest material of *Coptotermes gestroi* kindly provided by Prof. Dr. Rudy Plarre (Bundesanstalt für Materialforschung und –prüfung, Berlin). In brief, living cells were retrieved using nycodenz density gradient method, as described elsewhere [60]. Diluted cell suspensions (10^−2^–10^−6^) were plated on R2A Agar (DMSZ medium 830) and incubated for seven days at 28 °C. In order to isolate single strains, single colonies were propagated four times, then affiliated by using 16*S* rRNA gene sequencing with the primer pair E8F (5′-GAGTTTGATCCTGGCTCAG-3′) and 1492R (5′-AGAGTTTGATCCTGGCTCAG-3′) [61]. As judged on nearly full-length 16*S* rRNA sequence (MZ073637) comparison, FHG000416 is phylogenetically most closely related to *Olivibacter domesticus* DSM 18733 [48,49], with only ~94.5% identity.

### 4.2. Mass Spectrometric Analysis

For all UHPLC-QTOF-UHR-MS and MS/MS measurements, a quadrupole time-of-flight spectrometer (LC-QTOF maXis II, Bruker Daltonics, Bremen, Germany) equipped with an electrospray ionization source in line with an Agilent 1290 infinity LC system (Agilent Technologies, Santa Clara, CA, USA) was used. C18 RP-UHPLC (ACQUITY UPLC BEH C18 column (130 Å, 1.7 µm, 2.1 × 100 mm)) was performed at 45 °C with the following linear gradient (A: H_2_O, 0.1% HCOOH; B: CH_3_CN, 0.1% HCOOH; flow rate: 0.6 mL/min): 0 min: 95% A; 0.30 min: 95% A; 18.00 min: 4.75% A; 18.10 min: 0% A; 22.50 min: 0% A; 22.60 min: 95% A; 25.00 min: 95% A. A 50 to 2000 *m/z* scan range at 1 Hz scan rate was used to acquire mass spectral data. The injection volume was set to 5 μL. MS/MS experiments were performed at 6 Hz, and the top five most intense ions in each full MS spectrum were targeted for fragmentation by higher-energy collisional dissociation at 25 eV using N_2_ at 10^‒2^ mbar. Precursors were excluded after 2 spectra, released after 0.5 min, and reconsidered if the intensity of an excluded precursor increased by a factor of 1.5 or more. Data were analyzed using the Bruker Data Analysis 4.0 software package.

### 4.3. Isolation of Lipids

*C. eiseniae* DSM 22224 and *Olivibacter* sp. FHG000416 were inoculated from plate (R2A) in 300 mL Erlenmeyer flasks filled with 100 mL R2A and incubated at 28 °C with agitation at 180 rpm for 3 d. A 20 L fermentation (separated in 500 mL culture volume per 2 L flasks) of *C. eiseniae* in medium 3018 (1 g/L yeast extract, 5 g/L casitone, pH 7.0) was inoculated with 2% (*v*/*v*) pre-culture and incubated under the same conditions for 4 d. Using the same conditions, 7 and 20 L fermentations of *Olivibacter* sp. were performed in medium 5065 (15 g/L soluble starch, 10 g/L glucose, 10 g/L soy flour, 1 g/L yeast extract, 0.1 g/L K_2_HPO_4_, 3 g/L NaCl, pH 7.4) and 5294 (10 g/L soluble starch, 10 g/L glucose, 10 g/L glycerol 99%, 2.5 g/L liquid corn steep, 5 g/L peptone, 2 g/L yeast extract, 1 g/L NaCl, 3 g/L CaCO_3_, pH 7.2), respectively. An additional 40 L cultivation of *Olivibacter* sp. in medium 5294 was carried out using the same conditions as before. Cultures were subsequently lyophilized using a delta 2–24 LSCplus (Martin Christ Gefriertrockungsanlagen GmbH, Osterode am Harz, Germany).

The dried culture of *C. eiseniae* and the 7 L culture of *Olivibacter* sp. were extracted with one-time culture volume MeOH for the isolation of lipid **1**, lipid 430 (**2**), and **3**, respectively. The extracts were evaporated to dryness using rotary evaporation under reduced pressure, resuspended in 3 L of 10% MeOH/H_2_O, and separately loaded onto a XAD16N column (1 L bed volume). Step-wise elution with 10%, 40%, 60%, 80%, and 100% MeOH (2-times bed volume each) was performed. The 80% and 100% fractions containing the lipids were further fractionated by preparative (Synergi™ Fusion-RP 80 Å, 10 μm, 250 × 21.2 mm) and/or semi-preparative HPLC (Nucleodur^®^ C18 Gravity-SB, 3 μm, 250 × 10 mm) using gradients of 60–95% and 50–95% CH_3_CN (0.1% HCOOH) in water (0.1% HCOOH), respectively. Final purification was achieved by analytical HPLC (Synergi™ Fusion-RP 80 Å, 4 μm, 250 × 4.6 mm) or UHPLC fractionation (Acquity UPLC^®^ BEH C18, 1.7 μm, 100 × 2.1 mm) using a custom-made fraction collector (Zinsser–Analytik, Eschborn, Germany).

The 20 L fermentation of *Olivibacter* sp. was the starting point for the isolation of lipid **4**. Due to the enlarged volume, LLE was performed as an additional purification step after MeOH extraction using ethyl acetate and water. In addition to that, the isolation procedure was highly identical for lipid **3**. For semi-preparative HPLC, an adapted gradient of 55–95% CH_3_CN in water was used.

Extraction of the dried 40 L culture of *Olivibacter* sp. with MTBE/MeOH [62] was performed to isolate LPE 451 (**12**), **13**, and NAAAs **14**–**16**. Combined organic layers were subsequently fractionated by preparative and semi-preparative HPLC using gradients of 40–95% and 60–95% CH_3_CN (0.1% HCOOH) in water (0.1% HCOOH), respectively. Again, final purification was achieved by UHPLC fractionation. After each step, fractions containing compounds of interest were evaporated to dryness using a high performance evaporator (Genevac HT-12).

(2*S*)-5-Amino-2-((2*S*)-3-hydroxy-2-(2-(3-hydroxy-15 methylhexadecanamido)acetamido)propanamido)pentanoic acid (**1**). Colorless solid; ${\left[𝜶\right]}_{D}^{20.6}$ +21.1 (*c* 0.19, MeOH); LC-UV (CH_3_CN/H_2_O) λ_max_ 223 nm; ^1^H-NMR (500 MHz, DMSO-*d*_6_) data and 2D spectra, see Table 1, Figure S7–S10; UHRMS (ESI-TOF) *m/z* [M + H]^+^ calcd for C_27_H_52_N_4_O_7_^+^ 545.3909, found 545.3914.

Lipid 430 (**2**). Colorless solid;

$${\left[𝜶\right]}_{D}^{25.9}$$

+16.3 (*c* 0.37, MeOH); LC-UV (CH_3_CN/H_2_O) λ_max_ 220 nm; ^1^H-NMR (400 MHz, MeOD-*d*_4_) and ^13^C-NMR (101 MHz, MeOD-*d*_4_) data, see Table 1; UHRMS (ESI-TOF) *m/z* [M + H]^+^ calcd for C_22_H_42_N_2_O_6_^+^ 431.3116, found 431.3115.

(3-Hydroxy-15-methylhexadecanoyl)glycine (**3**). Colorless solid;

$${\left[𝜶\right]}_{D}^{21.7}$$

+66.7 (*c* 0.02, MeOH); LC-UV (CH_3_CN/H_2_O) λ_max_ 224 nm; ^1^H (600 MHz, DMSO-*d*_6_) and ^13^C-NMR (151 MHz, DMSO-*d*_6_) data, see Table 1, Figure S11–S15; UHRMS (ESI-TOF) *m/z* [M + H]^+^ calcd for C_19_H_37_NO_4_^+^ 344.2795, found 344.2802.

(2,3,4-Trihydroxy-15-methylhexadecanoyl)glycine (**4**). Colorless solid;

$${\left[𝜶\right]}_{D}^{21.7}$$

−76.9 (*c* 0.03, MeOH); LC-UV (CH_3_CN/H_2_O) λ_max_ 224 nm; ^1^H (600 MHz, DMSO-*d*_6_) and ^13^C-NMR (151 MHz, DMSO-*d*_6_) data, see Table S2, Figure S16–S20; UHRMS (ESI-TOF) *m/z* [M + H]^+^ calcd for C_19_H_37_NO_6_^+^ 376.2694, found 376.2696.

1-(9*Z*-Palmitoyl)-2-hydroxy-*sn*-glycerol-3-phosphoethanol-amine (**12**). Colorless solid; LC-UV (CH_3_CN/H_2_O) λ_max_ 223 nm; ^1^H (600 MHz, CDCl_3_/MeOD-*d*_4_ 2:1) and ^13^C-NMR (151 MHz, CDCl_3_/MeOD-*d*_4_ 2:1) data, see Table S3, Figure S21–S23; UHRMS (ESI-TOF) *m/z* [M + H]^+^ calcd for C_21_H_42_NO_7_P^+^ 452.2772, found 452.2769.

1-Isopentadecanoyl-2-hydroxy-*sn*-glycerol-3-phosphoethanolamine (**13**). Colorless solid; LC-UV (CH_3_CN/H_2_O) λ_max_ 224 nm; ^1^H (600 MHz, CDCl_3_/MeOD-*d*_4_ 2:1) and ^13^C-NMR (151 MHz, CDCl_3_/MeOD-*d*_4_ 2:1) data, see Table S3, Figure S24–S29; UHRMS (ESI-TOF) *m/z* [M + H]^+^ calcd for C_20_H_42_NO_7_P^+^ 440.2772, found 440.2767.

*N*-(5-Methyl)hexanoyl tyrosine (**14**). Yellowish solid; LC-UV (CH_3_CN/H_2_O) λ_max_ 224, 277 nm; ^1^H and ^13^C-NMR data, see Table 2, Figure S30–S34; UHRMS (ESI-TOF) *m/z* [M + H]^+^ calcd for C_16_H_23_NO_4_^+^ 294.1700, found 294.1698.

*N*-(7-Methyl)octanoyl tyrosine (**15**). Yellowish solid; LC-UV (CH_3_CN/H_2_O) λ_max_ 225, 277 nm; ^1^H and ^13^C-NMR data, see Table 2, Figure S35–S39; UHRMS (ESI-TOF) *m/z* [M + H]^+^ calcd for C_18_H_27_NO_4_^+^ 322.2013, found 322.2009.

*N*-(7-Methyl)octanoyl phenylalanine (**16**). Off-white solid; LC-UV (CH_3_CN/H_2_O) λ_max_ 217 nm; ^1^H and ^13^C-NMR data, see Table 2, Figure S40–S44; UHRMS (ESI-TOF) *m/z* [M + H]^+^ calcd for C_18_H_27_NO_3_^+^ 306.2064, found 306.2062.

#### NMR Studies

NMR spectra of LAA **1** were recorded on a Bruker AVANCE III 500 spectrometer (^1^H: 500 MHz, ^13^C: 125 MHz) equipped with a 10 mm MNP cryo probe. For all remaining compounds, NMR spectra were acquired on a Bruker AVANCE II/III HD 400 spectrometer (^1^H: 400 MHz, ^13^C: 101 MHz) or an AVANCE III HD 600 spectrometer (^1^H: 600 MHz, ^13^C: 151 MHz). Chemical shifts (*δ*) given in parts per million (ppm) are referenced to the residual solvent signals of DMSO-*d*_6_ (*δ*_H_ 2.50 and *δ*_C_ 39.5), CD_3_OD (*δ*_H_ 3.31 and *δ*_C_ 49.0), and CDCl_3_ (*δ*_H_ 7.26 and *δ*_C_ 77.2, in a 2:1 mixture with CD_3_OD).

### 4.4. Optical Rotation

Specific rotation was determined on a digital polarimeter (P3,000, A. Krüss Optronic GmbH). Standard wavelength was the sodium D-line with 589 nm. Temperature, concentration (g/100 mL), and solvents are reported with the determined value.

### 4.5. Advanced Marfey’s Analysis

The absolute configuration of all amino acids was determined by derivatization using Marfey’s reagent [50]. Stock solutions of amino acid standards (50 mM in H_2_O), NaHCO_3_ (1 M in H_2_O), and l-FDVA (70 mM in acetone) were prepared. Commercially available standards were derivatized using molar ratios of amino acid to FDVA and NaHCO_3_ (1/1.4/8). After stirring at 40 °C for 3 h, 1 M HCl was added to obtain a final concentration of 170 mM to end the reaction. Samples were subsequently evaporated to dryness and dissolved in DMSO (final concentration 50 mM). l- and d-amino acids were analyzed separately using C18 RP-UHPLC-MS with the standard gradient (for details see Section 4.2) at a flow rate of 0.6 mL/min.

Total hydrolysis of compounds **1**, lipid 430 (**2**), and **14**–**16** was carried out by dissolving 250 µg of each compound in 6 M deutero-hydrochloric acid (DCl in D_2_O) and stirring for 7 h at 160 °C. The sample was subsequently evaporated to dryness. Samples were dissolved in 100 µL H_2_O, derivatized, and analyzed using the same parameters as described before.

### 4.6. Minimal Inhibitory Concentration (MIC)

Microbroth dilution assays were performed in 96-well plates to determine the minimum inhibitory concentrations (MIC) of purified compounds dissolved in DMSO and were tested in triplicate following EUCAST instructions with minor adaptions [63,64]. A cell concentration of 5 × 10^5^ cells/mL was adjusted for all bacteria from an overnight culture (37 °C, 180 rpm) in cation-adjusted Mueller–Hinton II medium (BD). All tested organisms are summarized in Table 3. Dilution series of rifampicin, tetracycline, and gentamicin were used as control antibiotics (64–0.03 μg/mL) to ensure that concentrations achieved a range of effects from none to complete growth inhibition of the test strain. Negative controls were cell suspensions without test sample or antibiotic control. The turbidity was measured with a microplate spectrophotometer at 600 nm (LUMIstar Omega BMG Labtech) to assess cell growth after overnight incubation (18 h, 37 °C, 180 rpm, 85% rH).

*Mycobacterium smegmatis* ATCC 607 was grown in brain–heart infusion broth supplemented with Tween 80 (1.0% *v*/*v*) at 37 °C and 180 rpm for 48 h before the cell density was adjusted in cation-adjusted Mueller–Hinton II medium. The gentamicin control was replaced with isoniazid. Assay read out was done by cell viability assessment after 48 h (37 °C, 180 rpm, 85% rH) by ATP quantification (BacTiter-Glo, Promega), according to the manufacturer’s instructions.

*Candida albicans* FH2173 was incubated at 28 °C for 48 h before diluting to 1 × 10^6^ cells/mL in cation-adjusted Mueller–Hinton II medium. Assays were incubated at 37 °C for 48 h with nystatin as positive control and were evaluated by ATP quantification (BacTiter-Glo, Promega).

Pre- and main cultures of *Micrococcus luteus* DSM 20030 and *Listeria monocytogenes* DSM 20600 were incubated for two days, and the assay readout was done by ATP quantification, as described before.

## Figures and Tables

**Table 1:** ^1^H and ^13^C data of compounds **1**–**3** (**1**: 500 MHz/126 MHz, DMSO-*d*_6_; **2**: 400 MHz/101 MHz, MeOD-*d*_4_; **3:** 600 MHz/101 MHz, DMSO-*d*_6_).

	1		2		3	
Position	*δ*_H_, (*J* in Hz)	*δ*_C_, Type ^a^	*δ*_H_, (*J* in Hz)	*δ*_C_, Type	*δ*_H_, (*J* in Hz)	*δ*_C_, Type
1		171.1, C		175.0, C		171.1, C
2	2.20, d (6.1)	43.3, CH_2_	2.41, dd (14.0, 4.3), 2.34, dd (14.0, 8.6)	44.8, CH_2_	2.19, d (6.4)	43.6, CH_2_
3	3.82–3.75, m	67.2, CH	4.02–3.94, m	70.0, CH	3.79–3.74, m	67.4, CH
4	1.39–1.31, m	36.7, CH_2_	1.53–1.46, m	38.4, CH_2_	1.40–1.34, m, 1.33–1.27, m	36.8, CH_2_
5	1.39–1.31, m ^b^, 1.31–1.26, m ^b^	24.8, CH_2_	1.50–1.43, m, 1.38–1.32, m	26.7, CH_2_	1.40–1.35, m, 1.31–1.25, m	25.1, CH_2_
6	1.26–1.22, m ^b^	28.0–28.9 ^c^, CH_2_	1.36–1.25, m ^e^	30.7–31.0 ^f^, CH_2_	1.26–1.21, m ^g^	29.0–29.3 ^h^, CH_2_
7	1.26–1.22, m ^b^	28.0–28.9 ^c^, CH_2_	1.36–1.25, m ^e^	30.7–31.0 ^f^, CH_2_	1.26–1.21, m ^g^	29.0–29.3 ^h^, CH_2_
8	1.26–1.22, m ^b^	28.0–28.9 ^c^, CH_2_	1.36–1.25, m ^e^	30.7–31.0 ^f^, CH_2_	1.26–1.21, m ^g^	29.0–29.3 ^h^, CH_2_
9	1.26–1.22, m ^b^	28.0–28.9 ^c^, CH_2_	1.36–1.25, m ^e^	30.7–31.0 ^f^, CH_2_	1.26–1.21, m ^g^	29.0–29.3 ^h^, CH_2_
10	1.26–1.22, m ^b^	28.0–28.9 ^c^, CH_2_	1.36–1.25, m ^e^	30.7–31.0 ^f^, CH_2_	1.26–1.21, m ^g^	29.0–29.3 ^h^, CH_2_
11	1.26–1.22, m ^b^	28.0–28.9 ^c^, CH_2_	1.36–1.25, m ^e^	30.7–31.0 ^f^, CH_2_	1.26–1.21, m ^g^	29.0–29.3 ^h^, CH_2_
12	1.26–1.22, m ^b^	28.0–28.9 ^c^, CH_2_	1.36–1.25, m ^e^	30.7–31.0 ^f^, CH_2_	1.26–1.21, m ^g^	29.0–29.3 ^h^, CH_2_
13	1.26–1.22, m ^b^	26.4, CH_2_	1.36–1.25, m ^e^	28.5, CH_2_	1.26–1.21, m ^g^	26.8, CH_2_
14	1.16–1.11, m	38.1, CH_2_	1.17, q (6.7)	40.2, CH_2_	1.15–1.11, m	38.5, CH_2_
15	1.50, non (6.6)	27.0, CH	1.50, sept (6.6)	29.2, CH	1.49, non (6.6)	27.4, CH
16	0.85, d (6.6)	22.2, CH_3_	0.88, d (6.7)	23.0, CH_3_	0.84, d (6.6)	22.5, CH_3_
1′		168.6, C		171.6, C		n.o. ^d^
2′	3.73, d (4.6)	41.5, CH_2_	3.98, d (16.8), 3.90, d (16.7)	43.5, CH_2_	3.70, dd (17.4, 5.7), 3.65, dd (17.4, 5.7)	41.0, CH_2_
2-NH	8.10–8.04, m		8.30, t (5.6)		8.01, t (5.7)	
1′′′		n.o. ^d^		173.4, C		
2′′	4.25, dd (12.6, 6.2)	54.7, CH	4.50, t (4.3)	56.2, CH		
2′′-NH	7.91–7.83, m		8.04, d (7.7)			
3′′	3.56, m, 3.46, m	62.0, CH_2_	3.91, dd (11.5, 4.5), 3.83, dd (11.3, 3.8)	63.0, CH_2_		
1′′′		n.o. ^d^				
2′′′	3.82–3.75, m	53.6, CH				
2′′′-NH	7.66–7.59, m					
3′′′	1.80–1.71, m, 1.69–1.61, m	n.o. ^d^				
4′′′	1.66–1.56, m	24.0, CH_2_				
5′′′	2.80–2.73, m	38.4, CH_2_				

^a^ Extracted from HSQC spectra; ^b,c,e–h^ Signals are overlapping; ^d^ Not observed due to extreme line broadening.

**Table 2:** ^1^H and ^13^C data of compounds **14**–**16** (^1^H: 400 MHz, ^13^C: 101 MHz, MeOD-*d*_4_).

	14		15		16	
Position	*δ*_H_, (*J* in Hz)	*δ*_C_, Type	*δ*_H_, (*J* in Hz)	*δ*_C_, Type	*δ*_H_, (*J* in Hz)	*δ*_C_, Type
1′		157.3, C		157.2, C	7.23–7.17, m	127.8, CH
2′	6.69, dd (6.6, 1.9)	116.1, CH	6.68, dd (6.5, 2.1)	116.1, CH	7.30–7.22, m	129.4, CH
3′	7.04, dd (6.6, 1.9)	131.2, CH	7.03, dd (6.6, 1.5)	131.3, CH	7.26–7.21, m	130.3, CH
4′		129.2, C		129.4, C		138.7, C
5′	3.11, dd (14.0, 4.8), 2.83, dd (14.0, 9.3)	37.7, CH_2_	3.11, dd (14.0, 4.9), 2.84, dd (13.9, 8.9)	37.9, CH_2_	3.22, dd (13.9, 4.8), 2.93, dd (13.9, 9.5)	38.5, CH_2_
6′	4.59, dd (9.3, 4.6)	55.3, CH	4.57, dd (8.9, 4.9)	55.7, CH	4.66, dd (9.4, 4.8)	55.1, CH
7′		175.3, C		175.9, C		175.2, C
1		176.1, C		175.9, C		176.1, C
2	2.13, t (7.5)	37.1, CH_2_	2.15, t (7.4)	37.0, CH_2_	2.14, t (7.4)	36.9, CH_2_
3	1.55–1.47, m	24.8, CH_2_	1.56–1.48, m	27.0, CH_2_	1.55–1.46, m	26.9, CH_2_
4	1.14–1.05, m	39.4, CH_2_	1.32–1.24, m	28.2, CH_2_	1.32–1.23, m	28.2, CH_2_
5	1.52–1.46, m	29.0, CH	1.24–1.18, m	30.4, CH_2_	1.23–1.14, m	30.4, CH_2_
6	0.86, d (6.6)	22.8/22.9, CH_3_	1.20–1.12, m	40.0, CH_2_	1.22–1.10, m	40.0, CH_2_
7			1.57–1.49, m	29.1, CH	1.55–1.49, m	29.1, CH
8			0.88, d (6.6)	23.0, CH_3_	0.87, d (6.6)	23.1/23.0, CH_3_

**Table 3:** MIC values (µg/mL) of compounds **1**–**4** and **12**–**16**. MHC = cation-adjusted Mueller–Hinton II medium supplemented with 3.7 g/L bicarbonate, n.d. = not determined.

	Compounds								
	1	2	3	4	12	13	14	15	16
*E. coli* ATCC 35218 (MH-II)	>64	>64	>64	>64	>64	>64	>64	>64	>64
*E. coli* ATCC 35218 (MHC)	64	>64	>64	>64	>64	>64	>64	>64	>64
*E. coli* ATCC 25922 ΔTolC	>64	>64	>64	>64	>64	>64	>64	>64	>64
*P. aeruginosa* ATCC 27853	>64	>64	>64	>64	>64	>64	>64	>64	>64
*K. pneumoniae* ATCC 13883	>64	>64	>64	>64	>64	>64	>64	>64	>64
*M. catarrhalis* ATCC 25238	4–8	16–32	8	>64	4–8	16	64	>64	>64
*A. baumannii* ATCC 19606	n.d.	n.d.	>64	>64	n.d.	n.d.	>64	>64	>64
*B. subtilis* DSM 10	8	>64	>64	>64	>64	>64	>64	>64	>64
*S. aureus* ATCC 25923	>64	>64	>64	>64	>64	>64	>64	>64	>64
*M. luteus* DSM 20030	64	32–64	>64	>64	16	16	64	>64	>64
*L. monocytogenes* DSM 20600	n.d.	n.d.	>64	>64	>64	n.d.	>64	>64	>64
*M. smegmatis* ATCC 607	>64	n.d.	>64	n.d.	>64	n.d.	>64	>64	>64
*C. albicans* FH2173	64	>64	>64	>64	>64	>64	>64	>64	>64

## Data Availability

The 16*S* rRNA gene sequence of *Olivibacter* sp. FHG000416 is available at reference number MZ073637 (GenBank).

## Supplementary Materials

The following are available online, Figure S1: l-FDVA adducts of serine, Figure S2: l-FDVA adducts of ornithine, Figure S3: MS/MS spectra of NAAAs **14**–**16** (**A**–**C**) including postulated MS/MS fragmentation pathway, Figure S4: Double l-FDVA adducts of tyrosine, Figure S5: Integrated UV signals corresponding to double l-FDVA adducts of tyrosine for hydrolyzed NAAA **14** (top) and hydrolyzed NAAA **15** (bottom), Figure S6: l-FDVA adducts of phenylalanine, Figure S7: ^1^H-NMR (500 MHz, DMSO-*d*_6_) of lipid **1**, Figure S8: ^1^H-^1^H COSY (500 MHz, DMSO-*d*_6_) spectrum of lipid **1**, Figure S9: ^1^H-^13^C HSQC (500 MHz, DMSO-*d*_6_) spectrum of lipid **1**, Figure S10: ^1^H-^13^C HMBC (500 MHz, DMSO-*d*_6_) spectrum of lipid **1**, Figure S11: ^1^H-NMR (600 MHz, DMSO-*d*_6_) of lipid **3**, Figure S12: ^1^H-^1^H COSY (600 MHz, DMSO-*d*_6_) spectrum of lipid **3**, Figure S13: ^1^H-^13^C HSQC (600 MHz, DMSO-*d*_6_) spectrum of lipid **3**, Figure S14: ^1^H-^13^C HMBC (600 MHz, DMSO-*d*_6_) spectrum of lipid **3**, Figure S15: ^13^C-NMR (151 MHz, DMSO-*d*_6_) of lipid **3**, Figure S16: ^1^H-NMR (600 MHz, DMSO-*d*_6_) of lipid **4**, Figure S17: ^1^H-^1^H COSY (600 MHz, DMSO-*d*_6_) spectrum of lipid **4**, Figure S18: ^1^H-^13^C HSQC (600 MHz, DMSO-*d*_6_) spectrum of lipid **4**, Figure S19: ^1^H-^13^C HMBC (600 MHz, DMSO-*d*_6_) spectrum of lipid **4**, Figure S20: ^13^C-NMR (151 MHz, DMSO-*d*_6_) of lipid **4**, Figure S21: ^1^H-NMR (600 MHz, CDCl_3_/MeOD-*d*_4_ 2:1) of LPE 451 (**12**), Figure S22: ^13^C-NMR (151 MHz, CDCl_3_/MeOD-*d*_4_ 2:1) of LPE 451 (**12**), Figure S23: ^31^P-NMR (243 MHz, CDCl_3_/MeOD-*d*_4_ 2:1) of LPE 451 (**12**), Figure S24: ^1^H-NMR (600 MHz, CDCl_3_/MeOD-*d*_4_ 2:1) of LPE **13**, Figure S25: ^1^H-^1^H COSY (600 MHz, CDCl_3_/MeOD-*d*_4_ 2:1) of LPE **13**, Figure S26: ^1^H-^13^C HSQC (600 MHz, CDCl_3_/MeOD-*d*_4_ 2:1) of LPE **13**, Figure S27: ^1^H-^13^C HMBC (600 MHz, CDCl_3_/MeOD-*d*_4_ 2:1) of LPE **13**, Figure S28: ^13^C-NMR (151 MHz, CDCl_3_/MeOD-*d*_4_ 2:1) of LPE **13**, Figure S29: ^31^P-NMR (243 MHz, CDCl_3_/MeOD-*d*_4_ 2:1) of LPE **13**, Figure S30: ^1^H-NMR (400 MHz, MeOD-*d*_4_) of NAAA **14**, Figure S31: ^1^H-^1^H COSY (400 MHz, MeOD-*d*_4_) of NAAA **14**, Figure S32: ^1^H-^13^C HSQC (400 MHz, MeOD-*d*_4_) of NAAA **14**, Figure S33: ^1^H-^13^C HMBC (400 MHz, MeOD-*d*_4_) of NAAA **14**, Figure S34: ^13^C-NMR (101 MHz, MeOD-*d*_4_) of NAAA **14**, Figure S35: ^1^H-NMR (400 MHz, MeOD-*d*_4_) of NAAA **15**, Figure S36: ^1^H-^1^H COSY (400 MHz, MeOD-*d*_4_) of NAAA **15**, Figure S37: ^1^H-^13^C HSQC (400 MHz, MeOD-*d*_4_) of NAAA **15**, Figure S38: ^1^H-^13^C HMBC (400 MHz, MeOD-*d*_4_) of NAAA **15**, Figure S39: ^13^C-NMR (101 MHz, MeOD-*d*_4_) of NAAA **15**, Figure S40: ^1^H-NMR (400 MHz, MeOD-*d*_4_) of NAAA **16**, Figure S41: ^1^H-^1^H COSY (400 MHz, MeOD-*d*_4_) of NAAA **16**, Figure S42: ^1^H-^13^C HSQC (400 MHz, MeOD-*d*_4_) of NAAA **16**, Figure S43: ^1^H-^13^C HMBC (400 MHz, MeOD-*d*_4_) of NAAA **16**, Figure S44: ^13^C-NMR (101 MHz, MeOD-*d*_4_) of NAAA **16**, Table S1: Overview of all lipids with their molecular formula, predicted and found masses within the metabolomics data of our previous study, and in how many of the investigated 25 *Chitinophaga* metabolomes each lipid is present, Table S2: ^1^H and ^13^C data of compound **4** (^1^H: 600 MHz, ^13^C: 151 MHz, DMSO-*d*_6_), Table S3: ^1^H and ^13^C data of compounds **12** and **13** (^1^H: 600 MHz, ^13^C: 151 MHz, CDCl_3_/MeOD-*d*_4_ 2:1).

## Sample Availability

Samples of the compounds are not available from the authors.
