# Supplementary material for: Novel Glycerophospholipid, Lipo- and N-acyl Amino Acids from Bacteroidetes: Isolation, Structure Elucidation and Bioactivity
Source: Molecules. 2021 Aug 27;26(17):5195. doi: 10.3390/molecules26175195 (PMC8433624; doi:10.3390/molecules26175195)

molecules

Article

Supporting Information

Novel Glycerophospholipid, Lipo- and *N*-Acyl Amino Acids from Bacteroidetes: Isolation, Structure Elucidation and Bioactivity

Mona-Katharina Bill [1,†], Stephan Brinkmann [1,†], Markus Oberpaul [1], Maria A. Patras [1], Benedikt Leis [1], Michael Marner [1], Marc-Philippe Maitre [2], Peter E. Hammann [3,4], Andreas Vilcinskas [1,5], Sören M. M. Schuler [4,*] and Till F. Schäberle [1,5,*]

1 Fraunhofer Institute for Molecular Biology and Applied Ecology (IME), Branch for Bioresources, 35392 Giessen, Germany; mona.bill@ime.fraunhofer.de (M.-K.B.); stephan.brinkmann@ime.fraunhofer.de (S.B.); markus.oberpaul@ime.fraunhofer.de (M.O.); maria.patras@ime.fraunhofer.de (M.A.P.); benedikt.leis@gmx.net (B.L.); michael.marner@ime.fraunhofer.de (M.M.); andreas.vilcinskas@ime.fraunhofer.de (A.V.)
2 Sanofi Pasteur, R&D, 69280 Marcy L'Etoile, France; MarcPhilippe.Maitre@sanofi.com
3 Sanofi-Aventis Deutschland GmbH, R&D, 65926 Frankfurt am Main, Germany; peter.hammann@npconsult.me
4 Evotec International GmbH, 37079 Göttingen, Germany
5 Institute for Insect Biotechnology, Justus-Liebig-University of Giessen, 35392 Giessen, Germany
* Correspondence: soeren.schuler@evotec.com (S.M.M.S.); till.f.schaeberle@agrar.uni-giessen.de (T.F.S.)
† These authors contributed equally to this work.

Citation: Bill, M.-K.; Brinkmann, S.; Oberpaul, M.; Patras, M.A.; Leis, B.; Marner, M.; Maitre, M.-P.; Hammann, P.E.; Vilcinskas, A.; Schuler, S.M.M.; et al. Novel Glycerophospholipid, Lipo- and *N*-Acyl Amino Acids from Bacteroidetes: Isolation, Structure Elucidation and Bioactivity. *Molecules* **2021**, *26*, 5195. https://doi.org/10.3390/molecules26175195

Academic Editor: Natalizia Miceli

Received: 31 July 2021
Accepted: 25 August 2021
Published: 27 August 2021

Publisher's Note: MDPI stays neutral with regard to jurisdictional claims in published maps and institutional affiliations.

Copyright: © 2021 by the authors. Licensee MDPI, Basel, Switzerland. This article is an open access article distributed under the terms and conditions of the Creative Commons Attribution (CC BY) license (http://creativecommons.org/licenses/by/4.0/).

Keywords: linear lipoamino acid; lipid 430; lipid 654; *N*-acyl amino acid; lysophosphatidylethanolamine; bacteroidetes; *Chitinophaga*; *Olivibacter*; LC-MS/MS; antimicrobial lipids

Figure S1. L-FDVA adducts of serine. EICs (m/z 386.1306±0.005, $C_{14}H_{20}N_5O_8^+$, $[M+H]^+$) for **A**: D-serine reference, **B**: L-serine reference, **C**: hydrolyzed lipid 430 (**2**) and **D**: hydrolyzed lipid **1**.

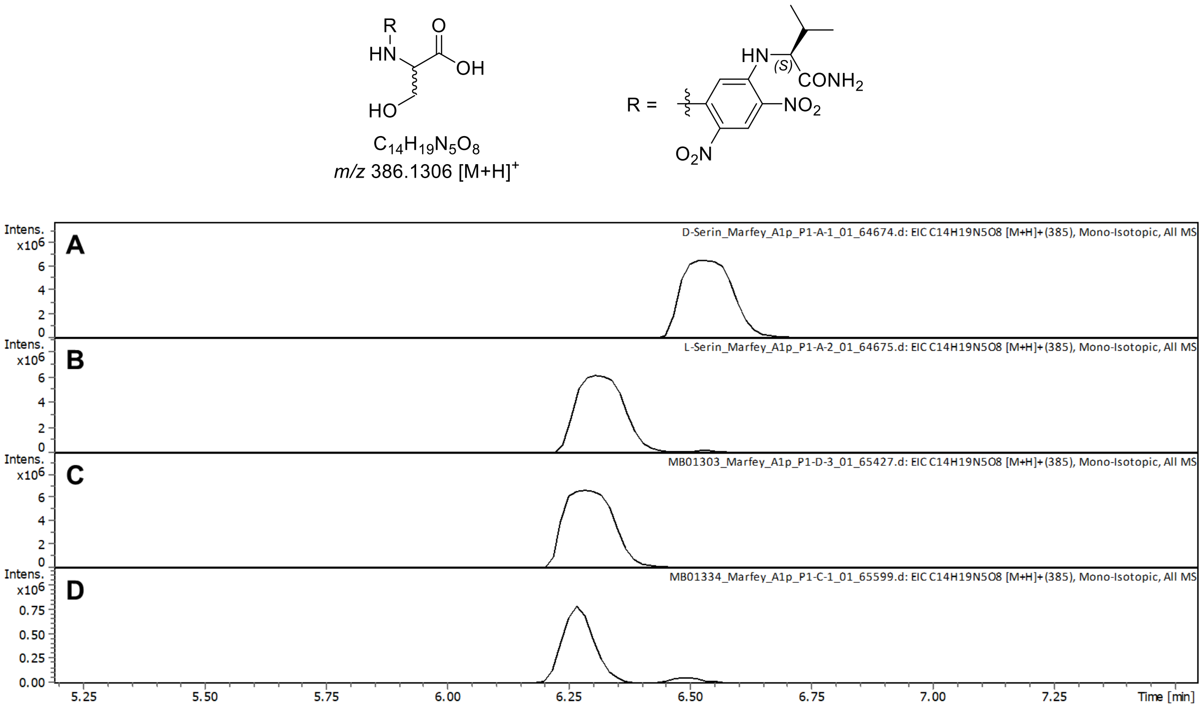

Figure S2. L-FDVA adducts of ornithine. EICs (m/z 413.1779±0.005, $C_{16}H_{25}N_6O_7^+$, $[M+H]^+$) for **A**: D-ornithine reference, **B**: L-ornithine reference, **C**: hydrolyzed lipid **1**.

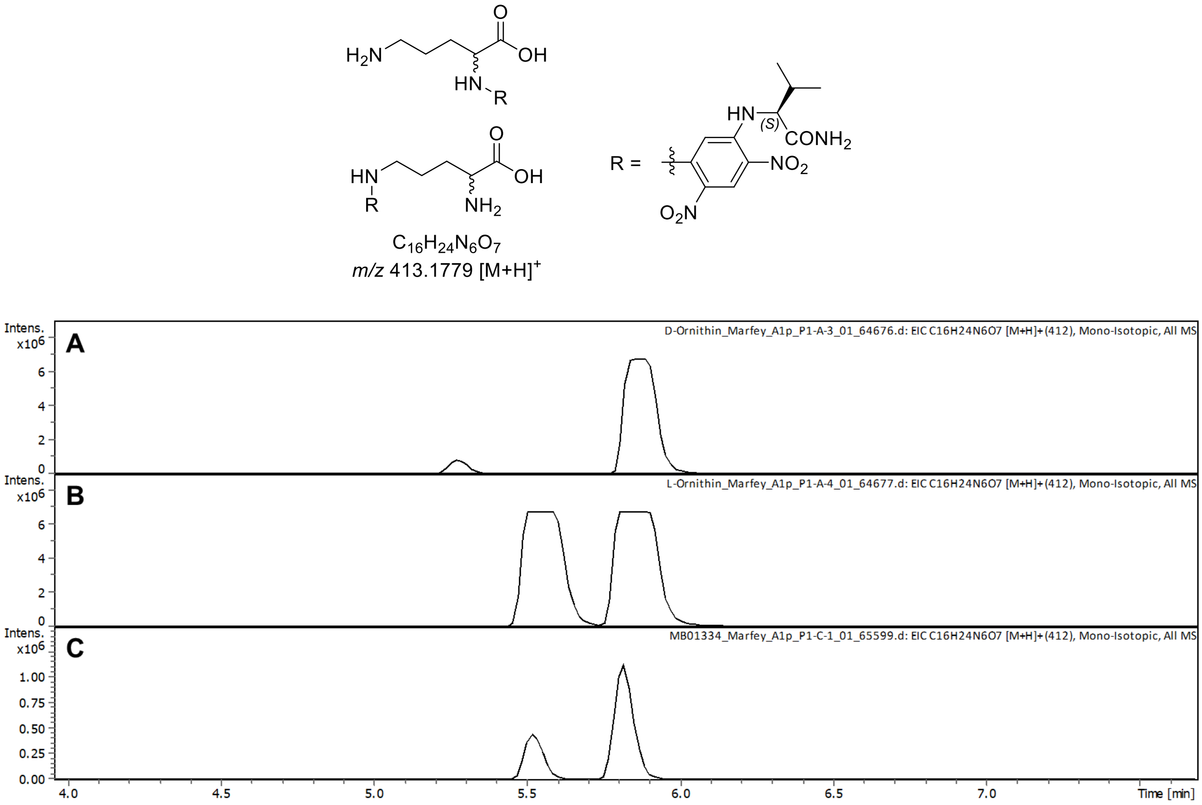

Table S1. Overview of all lipids with their molecular formula, predicted and found masses within the metabolomics data of our previous study, and in how many of the investigated 25 *Chitinophaga* metabolomes each lipid is present.

Compound	Molecular formula	Molecular mass [M+H]+			
		predicted	found	RT [s]	Presence in metabolomes
1	C27H52N4O7	545.3909	545.391	667.9	25
Lipid 430 (2)	C22H42N2O6	431.3116	431.312	778.5	23
Lipid 654	C37H70N2O7	655.5256	/	/	/
3	C19H37NO4	344.2795	344.280	834.1	22
4	C19H37NO6	376.2694	376.269	726.3	7
5	C42H80N4O8	769.6049	769.605	1020.6	8
6	C42H78N4O8	767.5892	767.589	1047.5	25
7	C26H50N4O7	531.3752	531.375	636.8	18
8	C42H80N4O9	785.5998	785.599	985.9	5
9	C41H78N4O9	771.5842	771.584	996.7	8
10	C40H76N4O9	757.5685	757.569	998.0	12
11	C41H78N4O8	755.5892	755.589	1050.2	4
LPE 451 (12)	C21H42NO7P	452.2772	452.277	704.7	25
13	C20H42NO7P	440.2772	440.277	720.3	25

Table S2. ^{1}H and ^{13}C data of compound **4** (^{1}H: 600 MHz, ^{13}C: 151 MHz, DMSO-d_6).

Position	4	
	δ_H, (*J* in Hz)	δ_C, type
1		171.3, C
2	4.19, s	70.9, CH
2-OH	5.25, br s	
3	3.44, dd (8.8, 1.1)	74.5, CH
3-OH	n.o. [a]	
4	3.40–3.35, m [b]	69.5, CH
4-OH	4.43, br s	
5	1.67–1.62, m,<br>1.29–1.19, m	33.4, CH_2
6	1.46–1.42, m,<br>1.29–1.19, m	25.1, CH_2
7	1.29–1.19, m [c]	29.3–29.0 [d], CH_2
8	1.29–1.19, m [c]	29.3–29.0 [d], CH_2
9	1.29–1.19, m [c]	29.3–29.0 [d], CH_2
10	1.29–1.19, m [c]	29.3–29.0 [d], CH_2
11	1.29–1.19, m [c]	29.3–29.0 [d], CH_2
12	1.29–1.19, m [c]	29.3–29.0 [d], CH_2
13	1.29–1.19, m [c]	26.8, CH_2
14	1.15–1.12, m	38.5, CH_2
15	1.49, non (6.6)	27.4, CH
16	0.84, d (6.6)	22.5, CH_3
1′		173.8, C
2′	3.67, dd (17.6, 5.3),<br>3.80, dd (17.4, 5.8)	41.0, CH_2
2′-NH	7.76, t (5.6)	

[a] n.o.: Not observed; [b] Extracted from HSQC spectra due to overlapping water signal; [c,d] Signals are overlapping

Table S3. ^1H and ^{13}C data of compounds **12** and **13** (^1H: 600 MHz, ^{13}C: 151 MHz, $CDCl_3$/MeOD-d_4 2:1).

	**12**			**13**		
**Position**	**δ_H, (*J* in Hz)**	**δ_C, type**	**δ_P**	**δ_H, (*J* in Hz)**	**δ_C, type**	**δ_P**
1′′	3.01, br s	40.3, CH_2		2.91, br s	40.3, CH_2	
2′′	3.99–3.92, m [a]	61.6, CH_2		3.87–3.83, m [a]	61.3, CH_2	
3′′			4.42			0.75
1′	3.84–3.70, m [a]	66.9, CH_2		3.74–3.63, m [a]	66.7, CH_2	
2′	3.88–3.82, m [a]	68.5, CH		3.79–3.74, m [a]	68.5, CH	
3′	4.01–3.93, m [a]	64.7, CH_2		3.93–3.88, m [a]	64.6, CH_2	
1		174.2, C			174.1, C	
2	2.19, t (7.5)	33.9, CH_2		2.12, t (7.9)	33.8, CH_2	
3	1.51–1.43, m	24.7, CH_2		1.42–1.36, m	24.6, CH_2	
4	1.22–1.10, m [b]	31.6–28.8 [e], CH_2		1.11–1.01, m [g]	29.6–27.1 [h], CH_2	
5	1.22–1.10, m [b]	31.6–28.8 [e], CH_2		1.11–1.01, m [g]	29.6–27.1 [h], CH_2	
6	1.22–1.10, m [b]	31.6–28.8 [e], CH_2		1.11–1.01, m [g]	29.6–27.1 [h], CH_2	
7	1.22–1.10, m [b]	31.6–28.8 [e], CH_2		1.11–1.01, m [g]	29.6–27.1 [h], CH_2	
8	1.91–1.83, m [c]	27.0, CH_2		1.11–1.01, m [g]	29.6–27.1 [h], CH_2	
9	5.24–5.12, m [d]	129.9 [f], CH		1.11–1.01, m [g]	29.6–27.1 [h], CH_2	
10	5.24–5.12, m [d]	129.6 [f], CH		1.11–1.01, m [g]	29.6–27.1 [h], CH_2	
11	1.91–1.83, m [c]	27.0, CH_2		1.11–1.01, m [g]	29.6–27.1 [h], CH_2	
12	1.22–1.10, m [b]	31.6–28.8 [e], CH_2		0.95–0.90, m	38.8, CH_2	
13	1.22–1.10, m [b]	31.6–28.8 [e], CH_2		1.29, non (6.7)	27.7, CH	
14	1.22–1.10, m [b]	31.6–28.8 [e], CH_2		0.63, d (6.6)	22.2, CH_3	
15	1.18–1.11, m [a]	22.5, CH_2				
16	0.73, t (6.9)	13.8, CH_3				

[a] Extracted from HSQC spectra; [b-e,g,h] Signals are overlapping; [f] Might be interchangeable.

Figure S3. MS/MS spectra of NAAAs **14–16** (**A–C**) including postulated MS/MS fragmentation pathway.

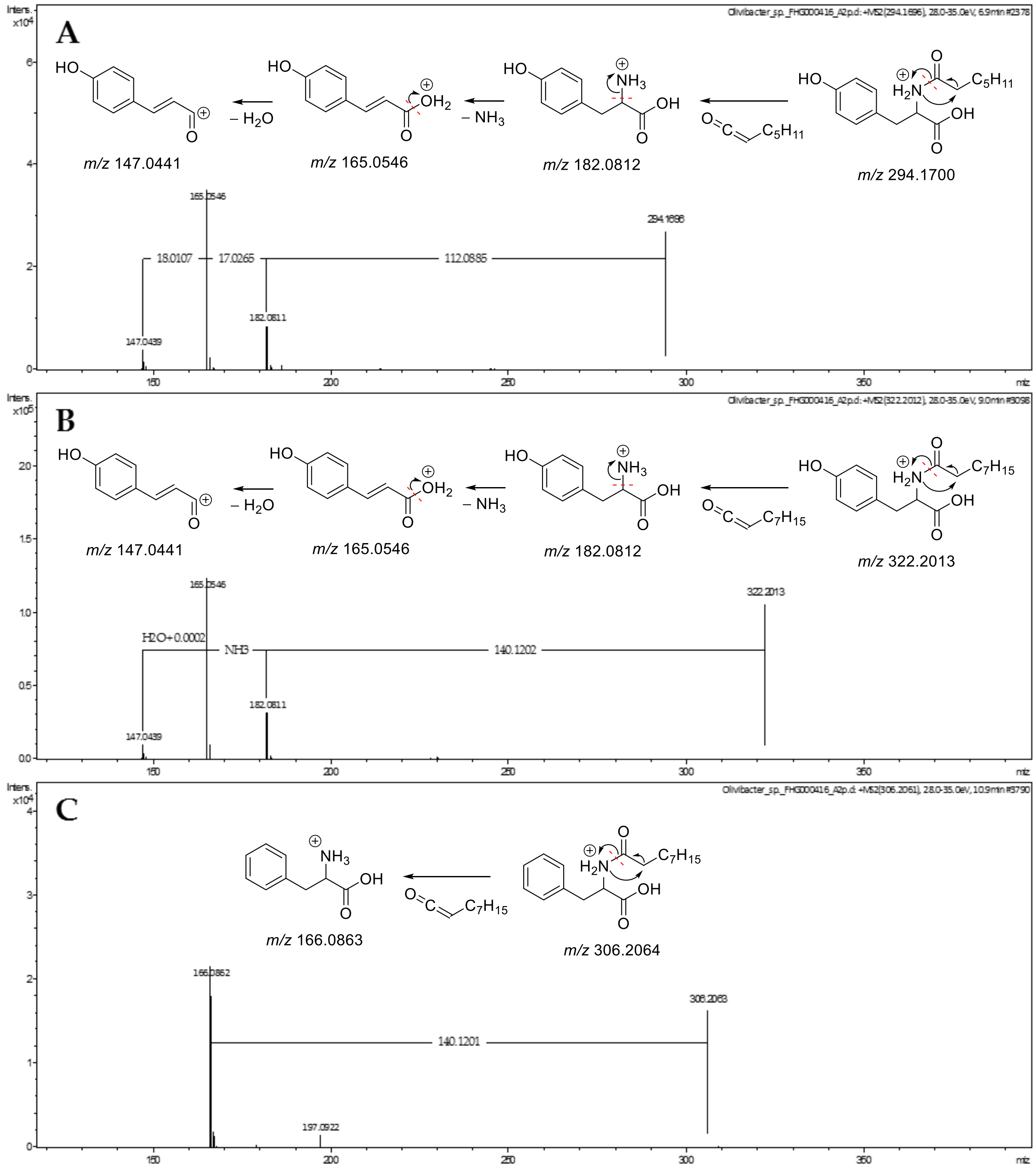

Figure S4. Double L-FDVA adducts of tyrosine. EICs (*m/z* 742.2427±0.005, $C_{31}H_{36}N_9O_{13}^+$, $[M+H]^+$) for **A**: D-tyrosine reference, **B**: L-tyrosine reference and EICs (*m/z* 744.2553±0.005, $C_{31}H_{34}D_2N_9O_{13}^+$, $[M+H]^+$) for **C**: hydrolyzed NAAA **14** and **D**: hydrolyzed NAAA **15**. Twofold hydrogen-deuterium exchange in benzylic position of tyrosine was observed after hydrolysis in DCl/D_2O.

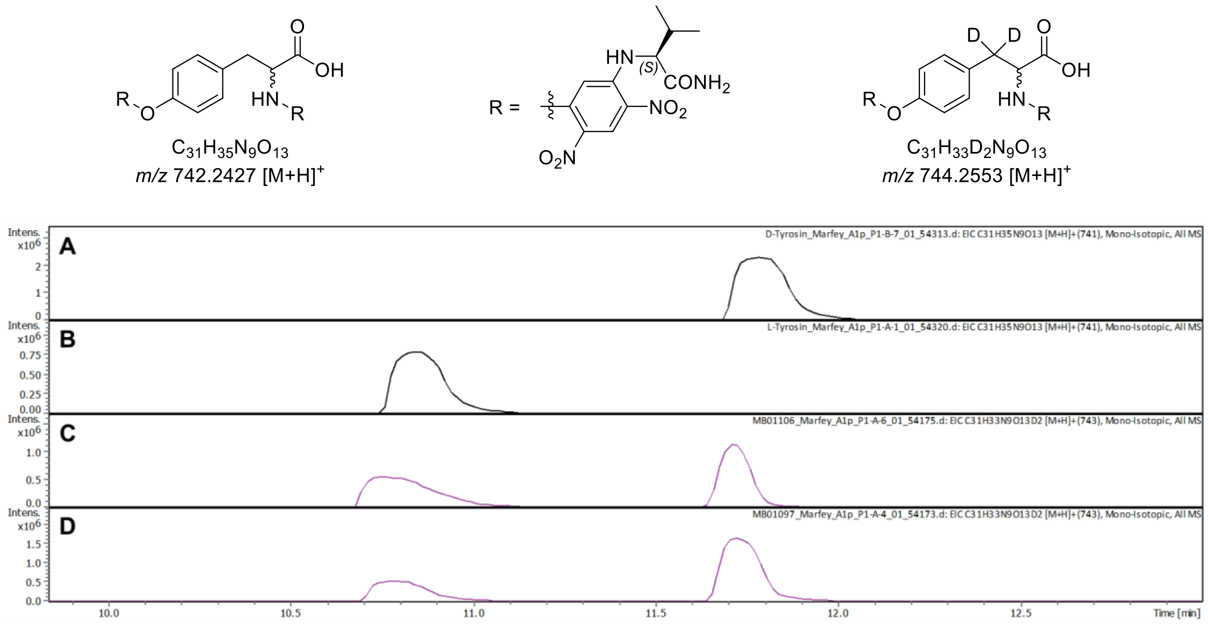

Figure S5. Integrated UV signals corresponding to double L-FDVA adducts of tyrosine for hydrolyzed NAAA **14** (top) and hydrolyzed NAAA **15** (bottom).

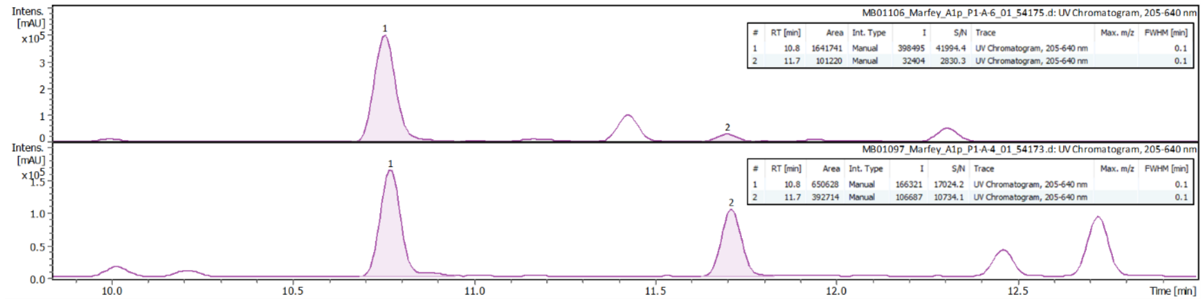

Figure S6. L-FDVA adducts of phenylalanine. EICs (*m/z* 446.1670±0.005, $C_{20}H_{24}N_5O_7^+$, $[M+H]^+$) for **A**: L-phenylalanine reference, **B**: DL-phenylalanine reference and **C**: hydrolyzed NAAA **16** (including integrated UV signals).

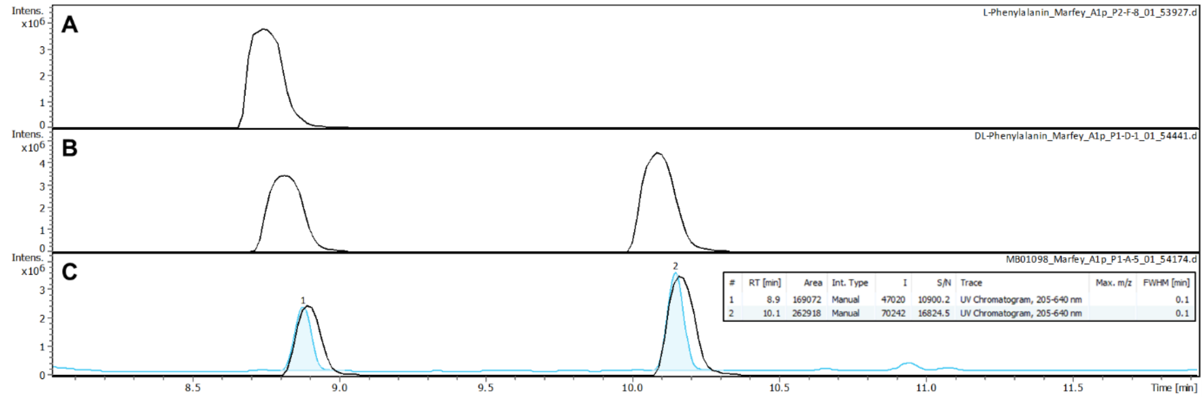

Figure S7. ^{1}H-NMR (500 MHz, DMSO-d_6) of lipid **1**.

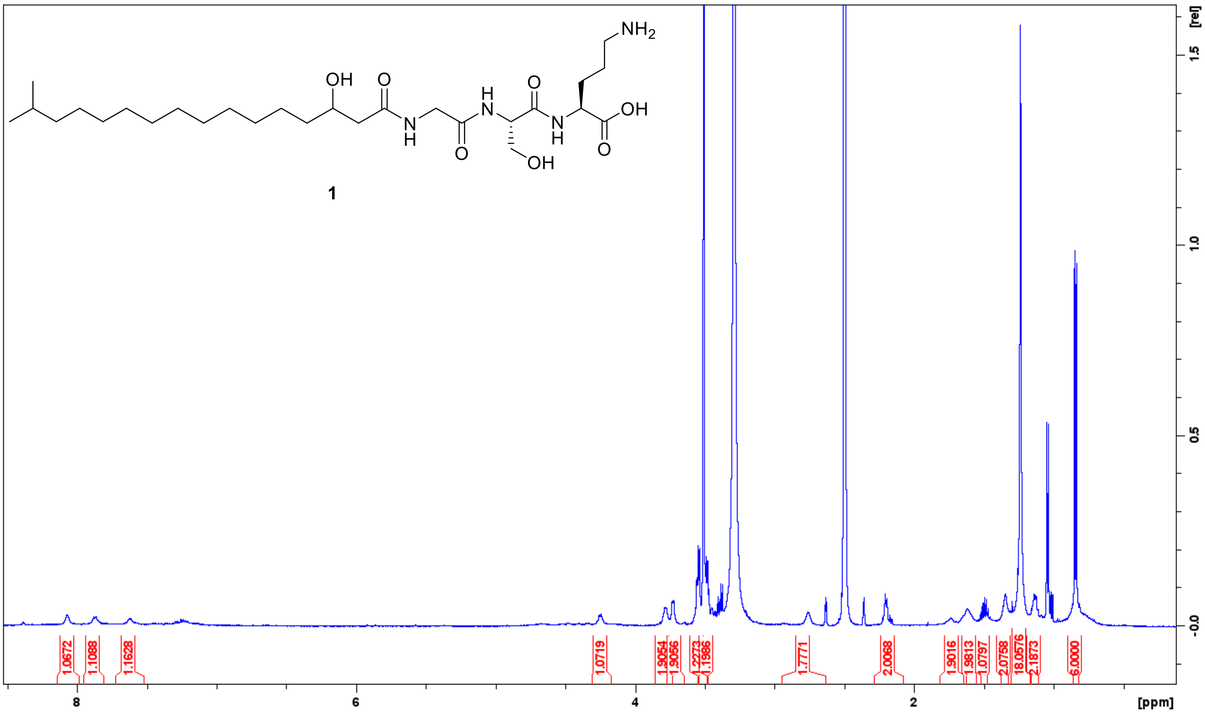

Figure S8. ^{1}H-^{1}H COSY (500 MHz, DMSO-d_6) spectrum of lipid **1**.

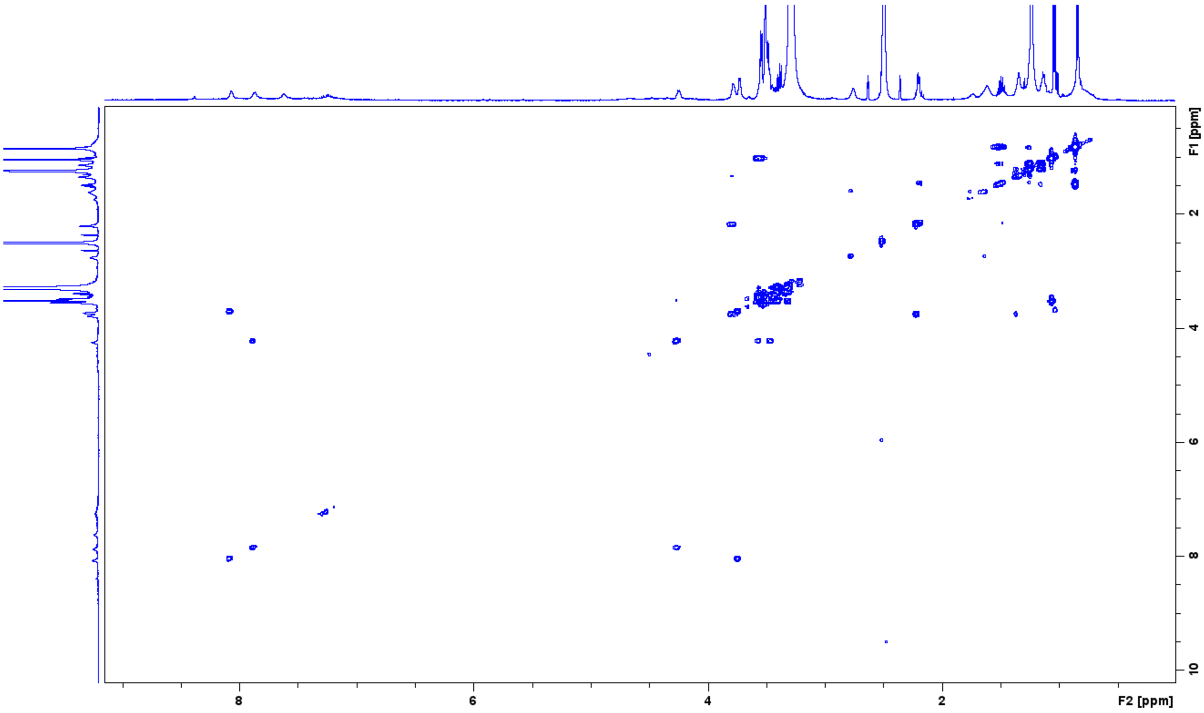

Figure S9. ^{1}H-^{13}C HSQC (500 MHz, DMSO-d_6) spectrum of lipid **1**.

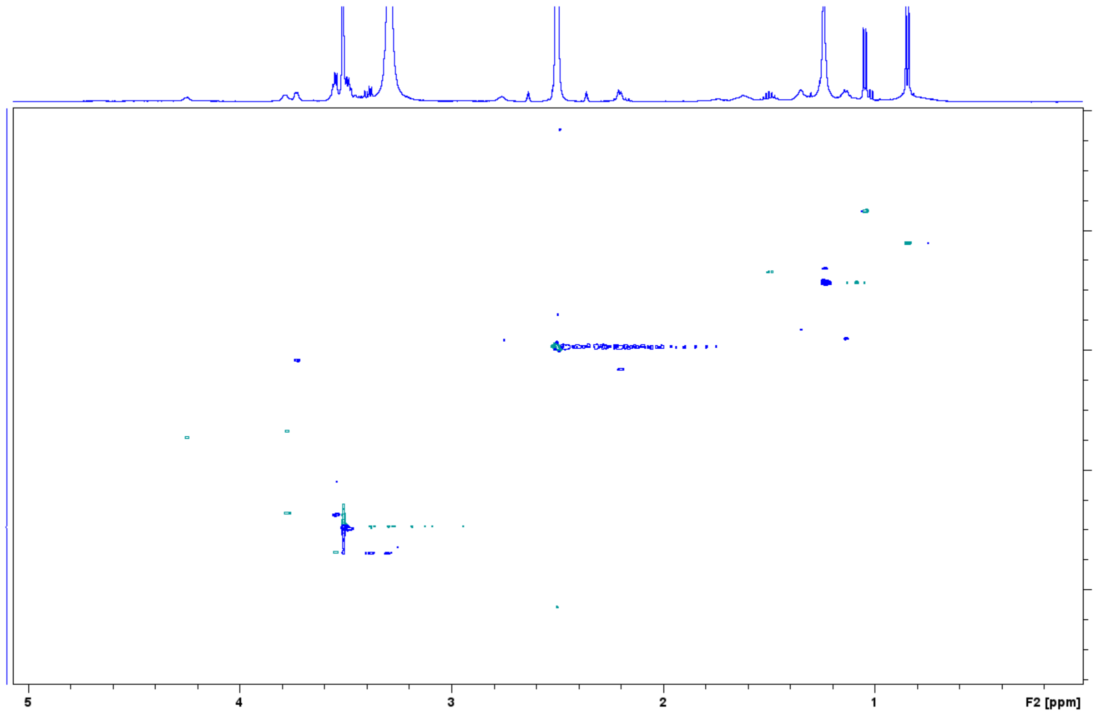

Figure S10. ^{1}H-^{13}C HMBC (500 MHz, DMSO-d_6) spectrum of lipid **1**.

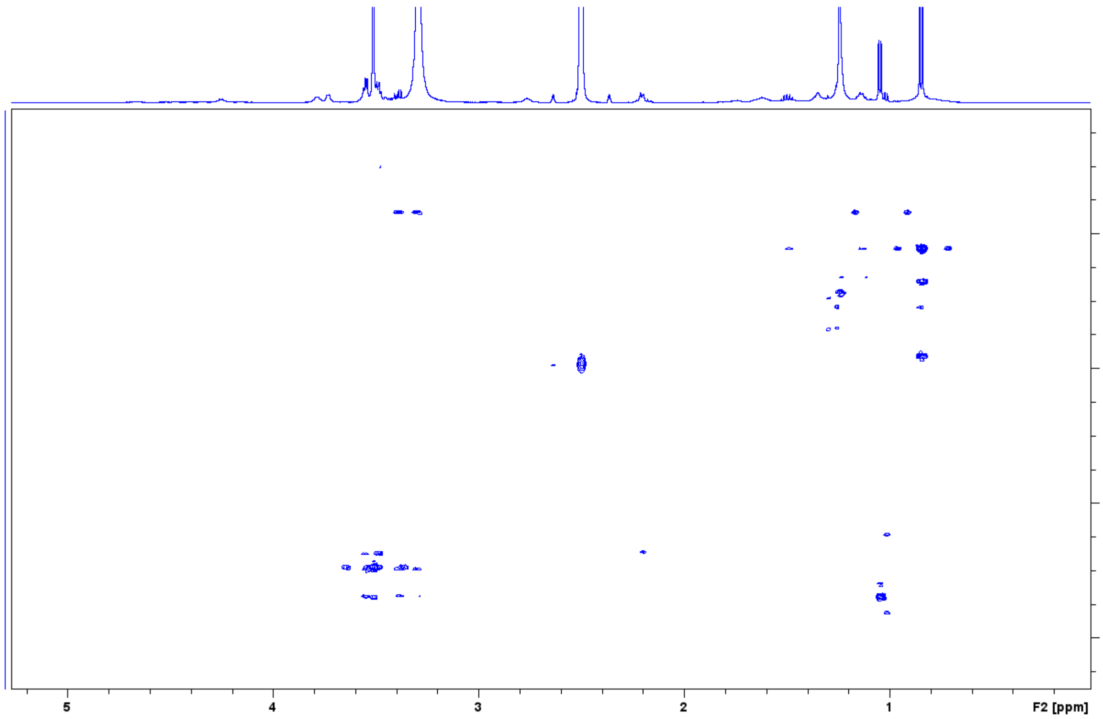

Figure S11. ^{1}H-NMR (600 MHz, DMSO-d_6) of lipid **3**.

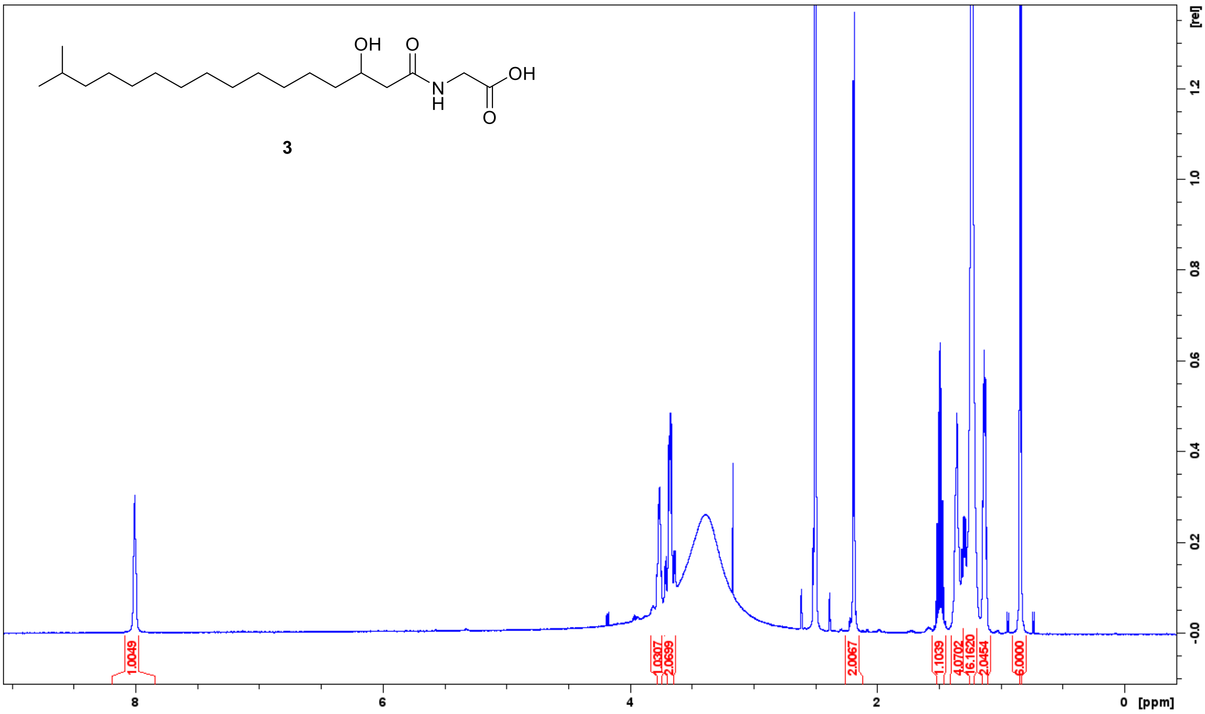

Figure S12. ^{1}H-^{1}H COSY (600 MHz, DMSO-d_6) spectrum of lipid **3**.

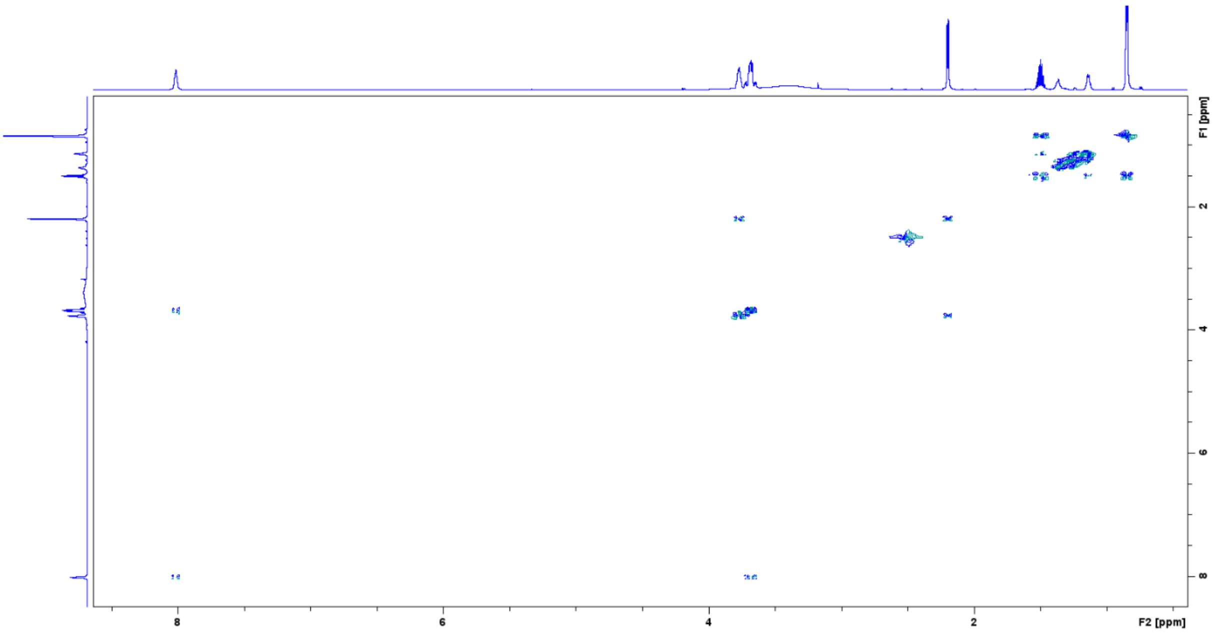

Figure S13. ^{1}H-^{13}C HSQC (600 MHz, DMSO-d_6) spectrum of lipid **3**.

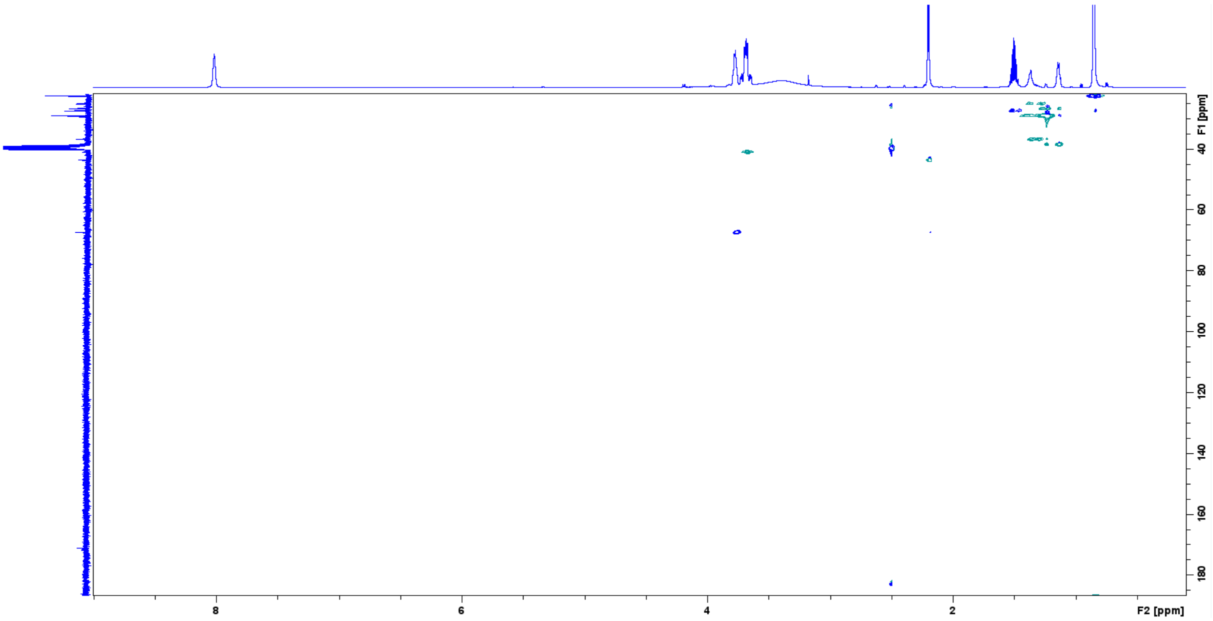

Figure S14. ^{1}H-^{13}C HMBC (600 MHz, DMSO-d_6) spectrum of lipid **3**.

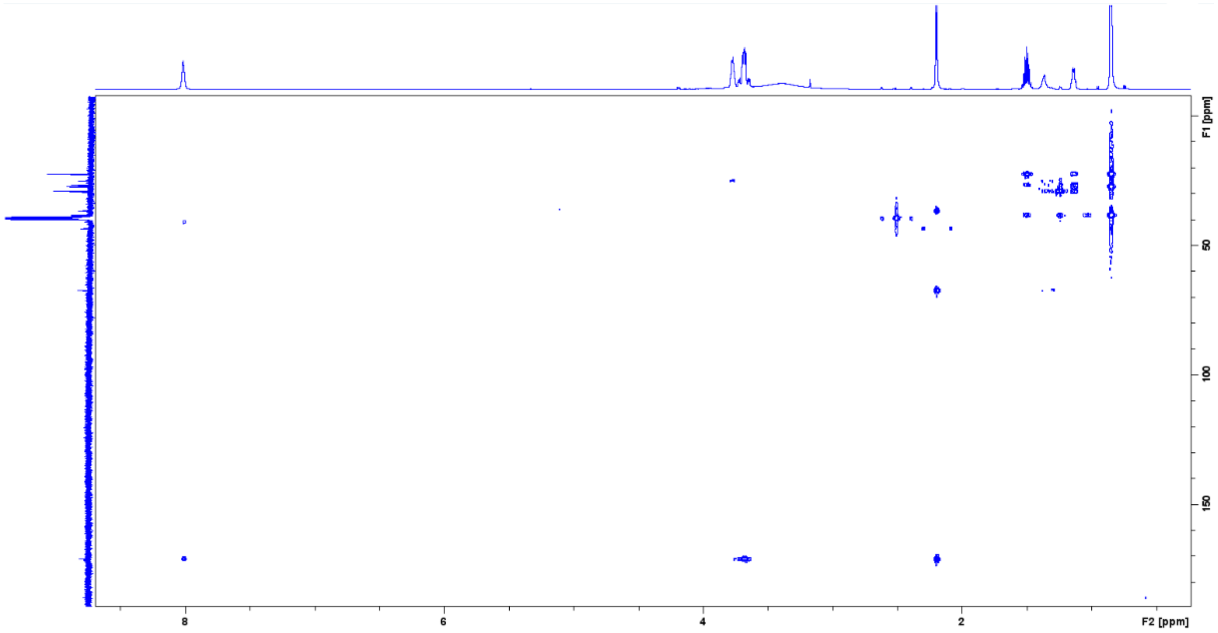

Figure S15. ^{13}C-NMR (151 MHz, DMSO-d_6) of lipid **3**.

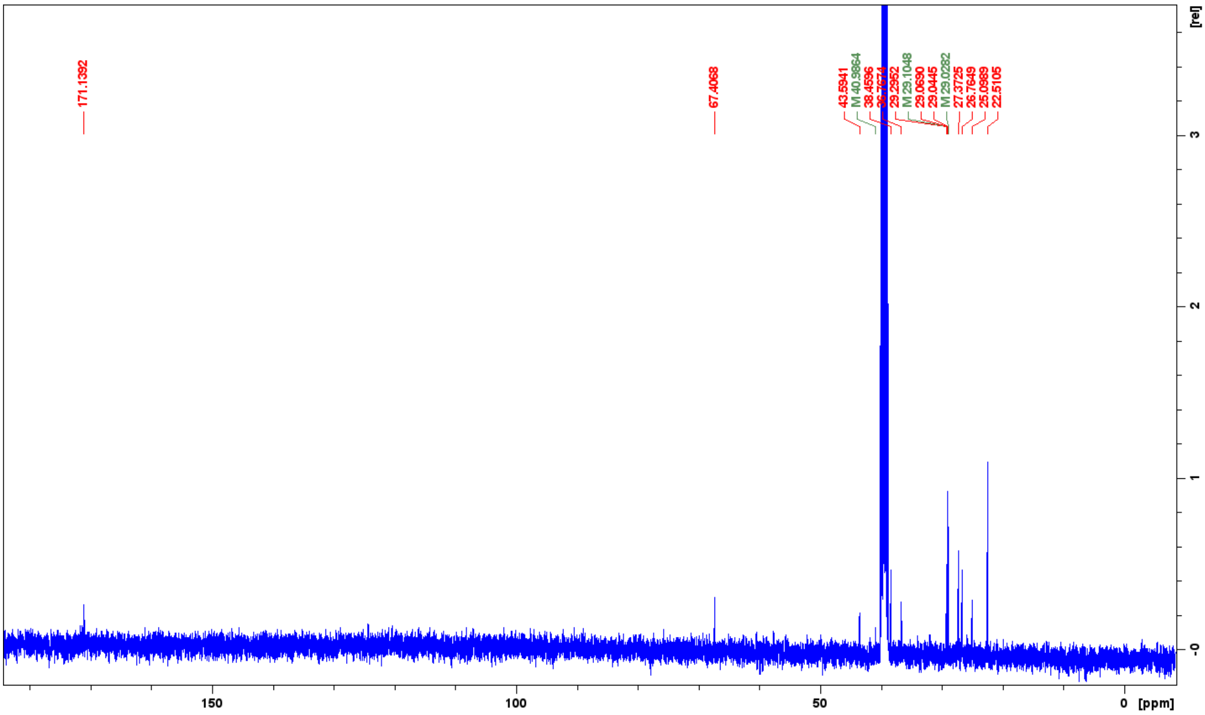

Figure S16. ^{1}H-NMR (600 MHz, DMSO-*d*6) of lipid **4**.

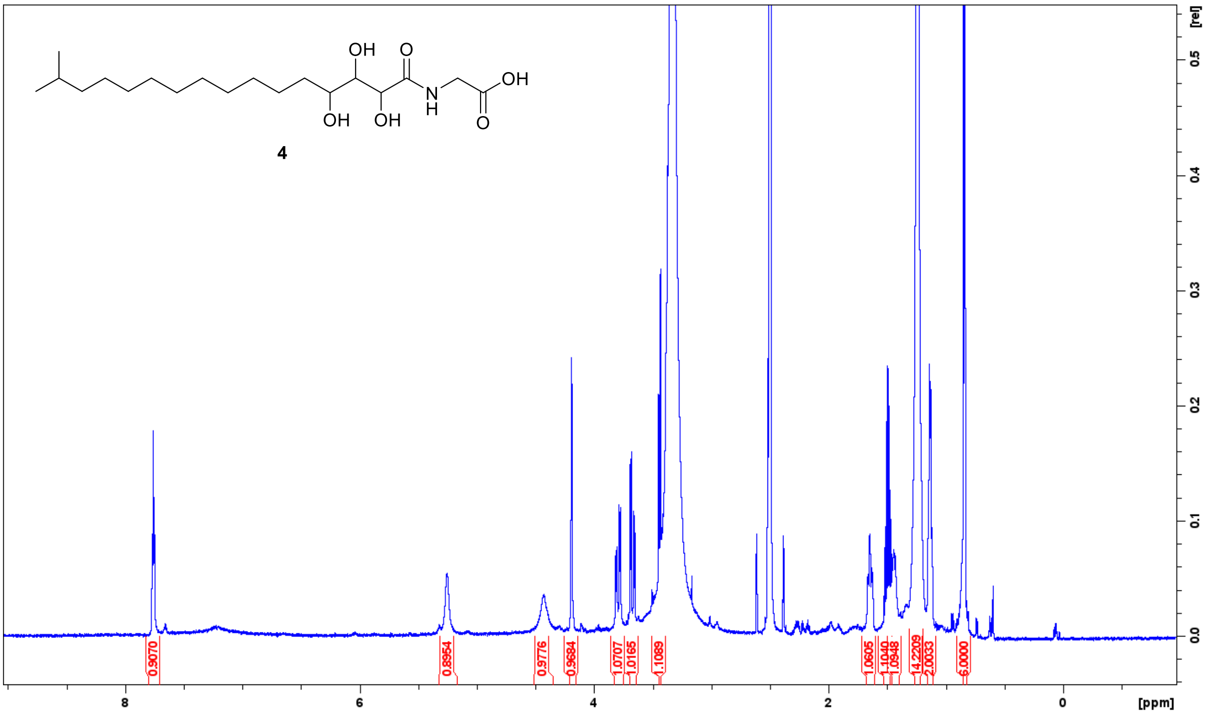

Figure S17. ^{1}H-^{1}H COSY (600 MHz, DMSO-*d*6) spectrum of lipid **4**.

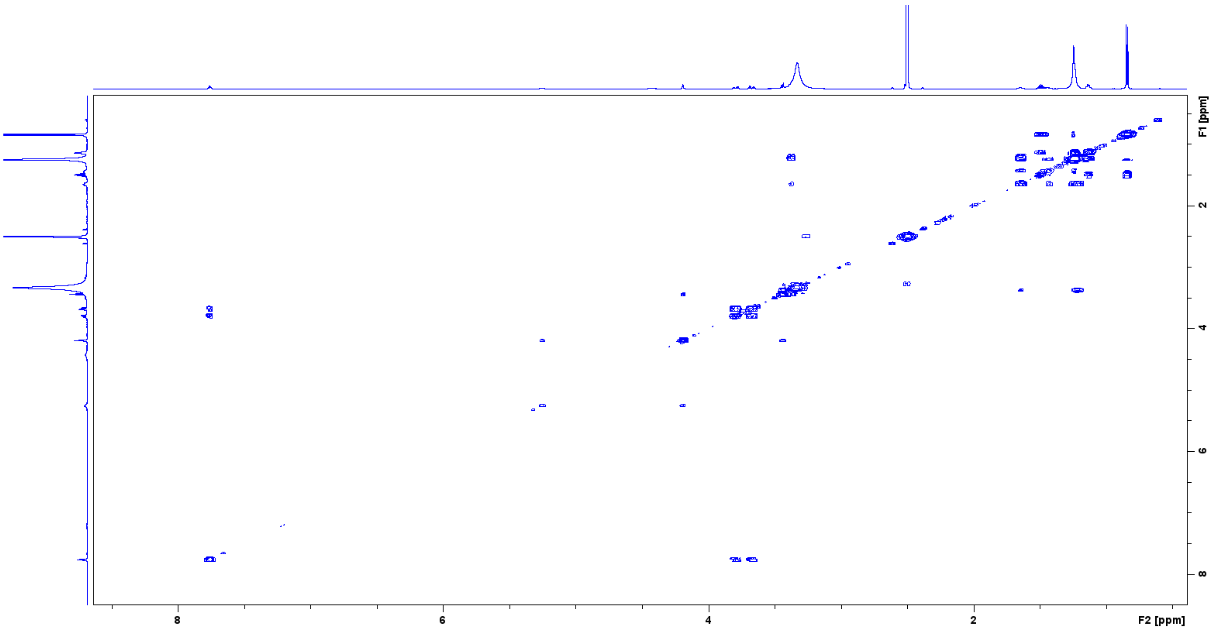

Figure S18. ^{1}H-^{13}C HSQC (600 MHz, DMSO-d_6) spectrum of lipid **4**.

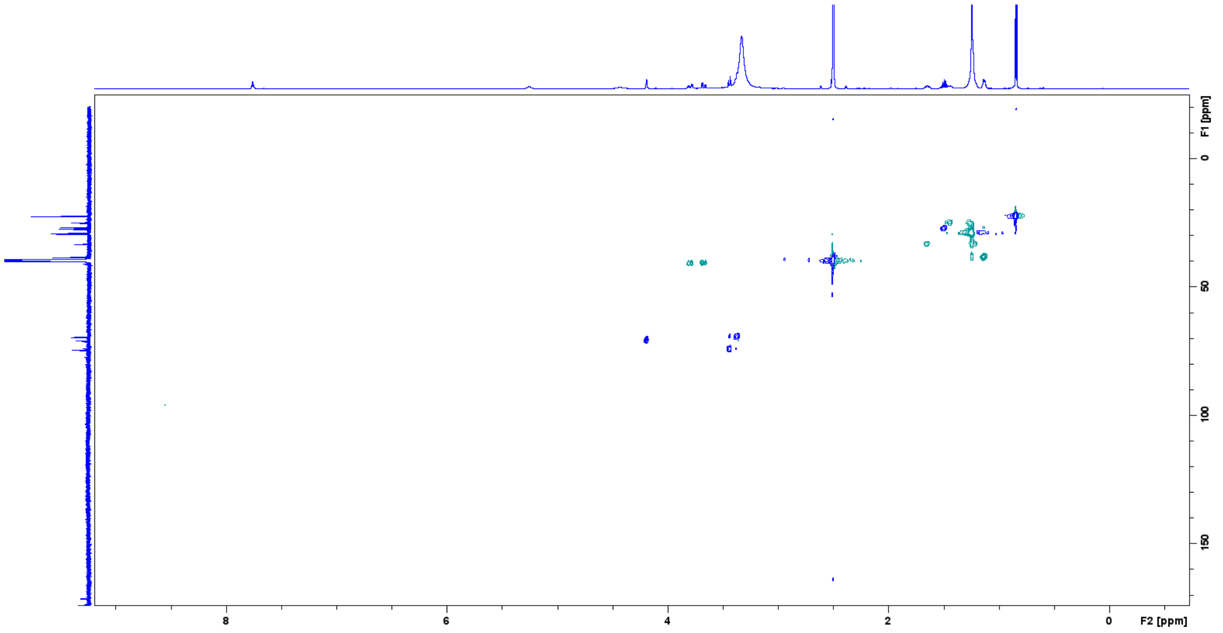

Figure S19. ^{1}H-^{13}C HMBC (600 MHz, DMSO-d_6) spectrum of lipid **4**.

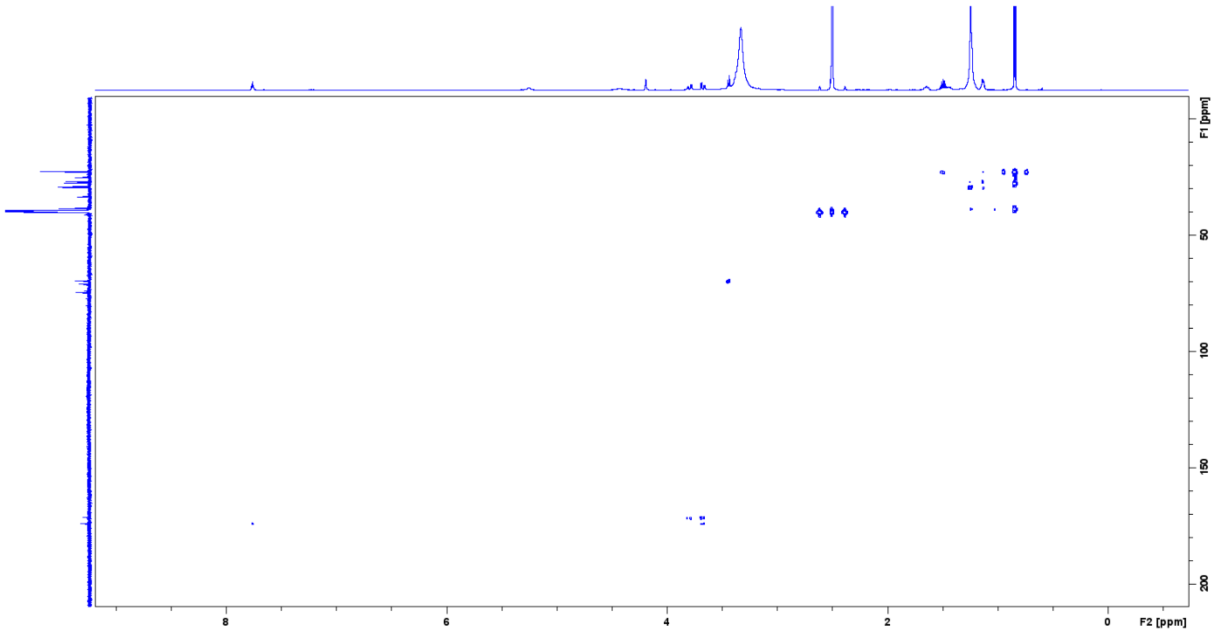

Figure S20. ^{13}C-NMR (151 MHz, DMSO-*d*$_6$) of lipid **4**.

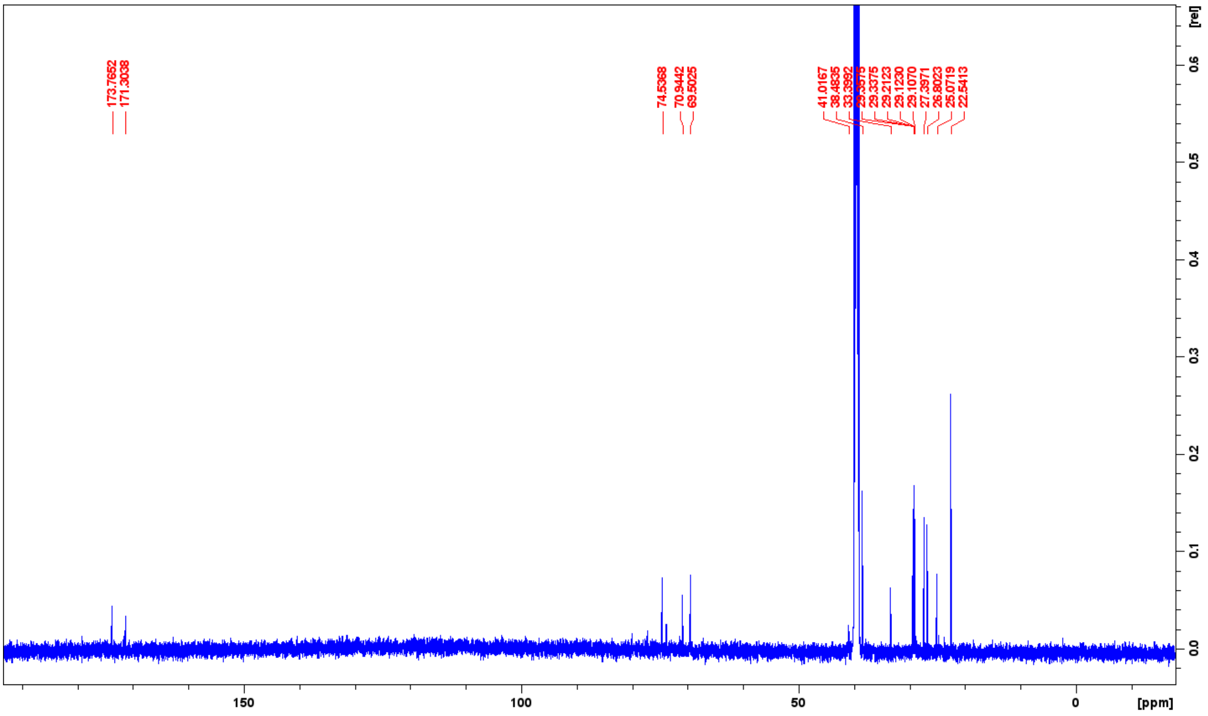

Figure S21. ^{1}H-NMR (600 MHz, $CDCl_3$/MeOD-*d*$_4$ 2:1) of LPE 451 (**12**).

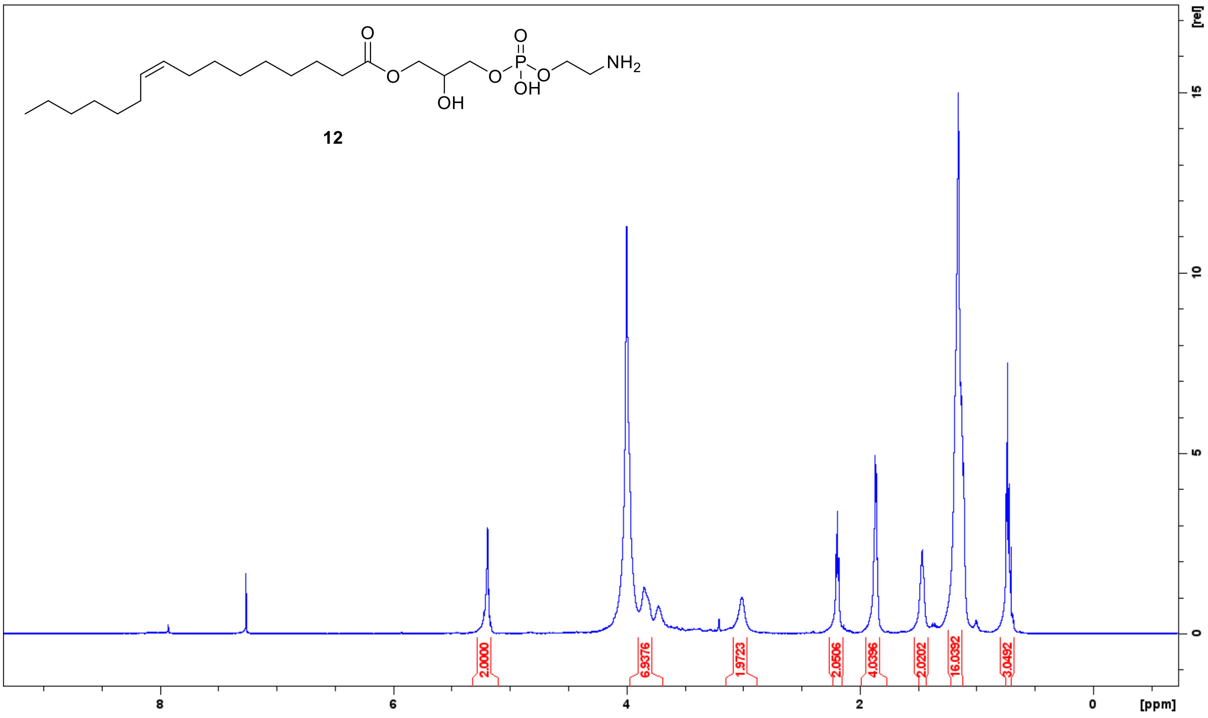

Figure S22. ^{13}C-NMR (151 MHz, $CDCl_3$/MeOD-d_4 2:1) of LPE 451 (**12**).

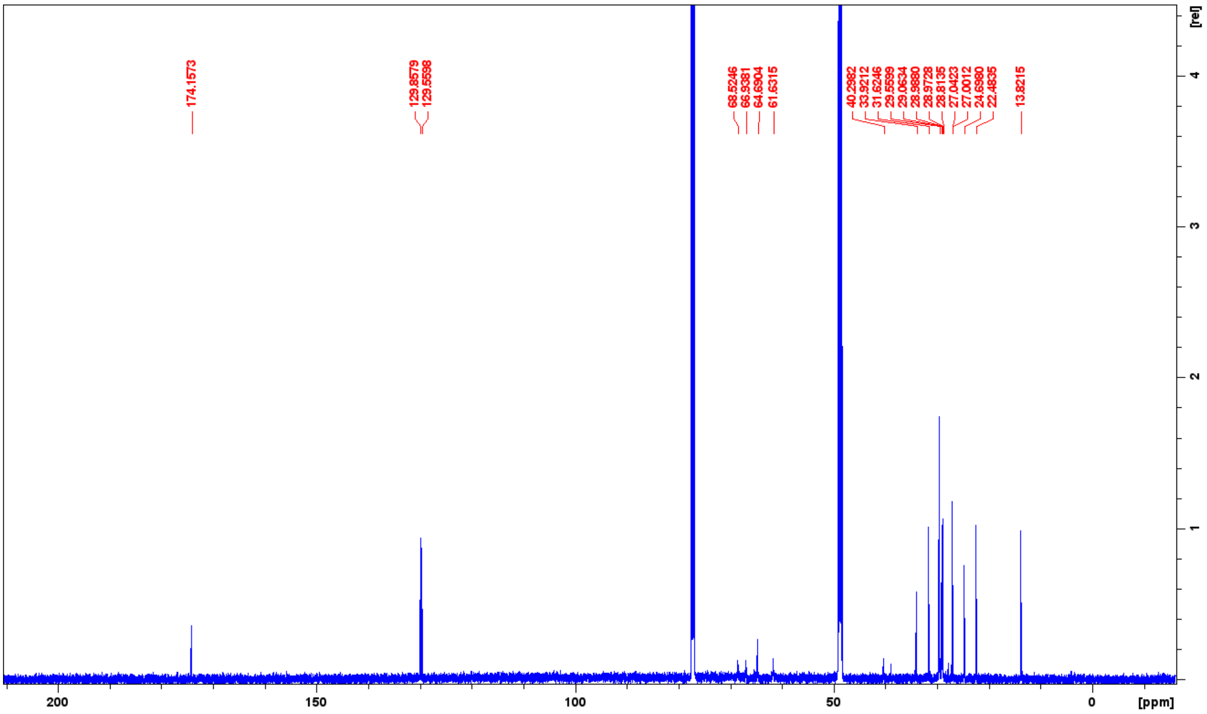

Figure S23. ^{31}P-NMR (243 MHz, $CDCl_3$/MeOD-d_4 2:1) of LPE 451 (**12**).

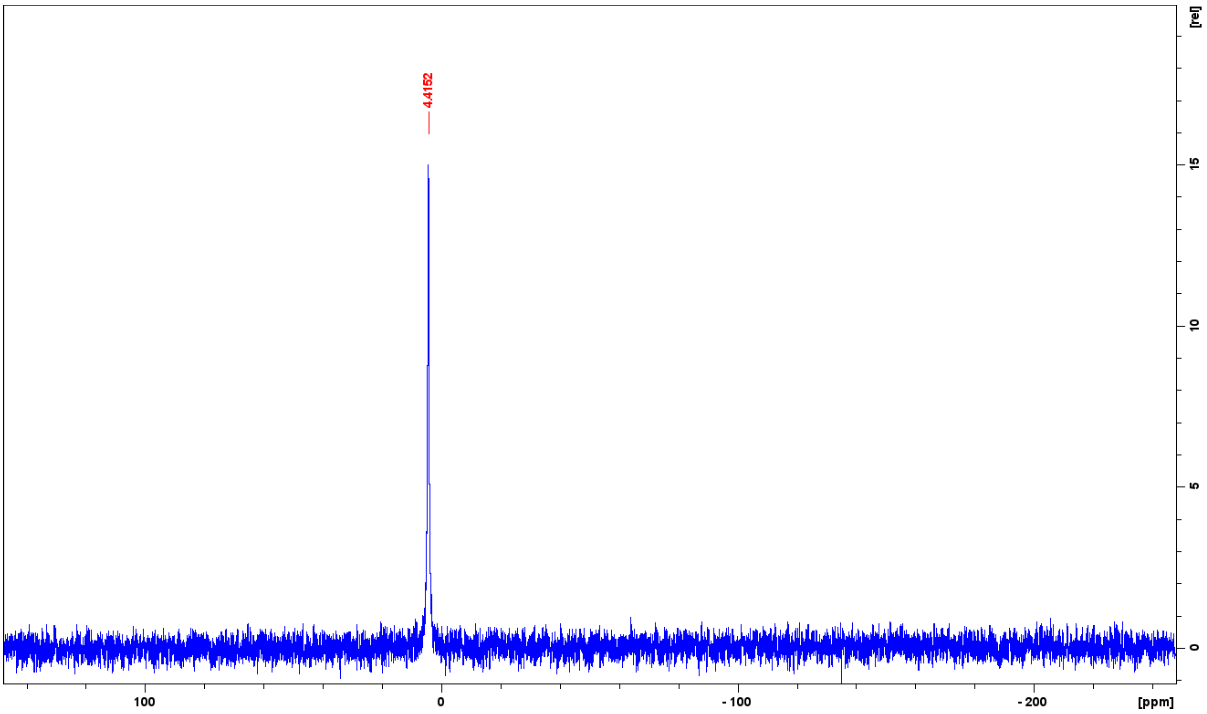

Figure S24. ^{1}H-NMR (600 MHz, $CDCl_3$/MeOD-d_4 2:1) of LPE **13**.

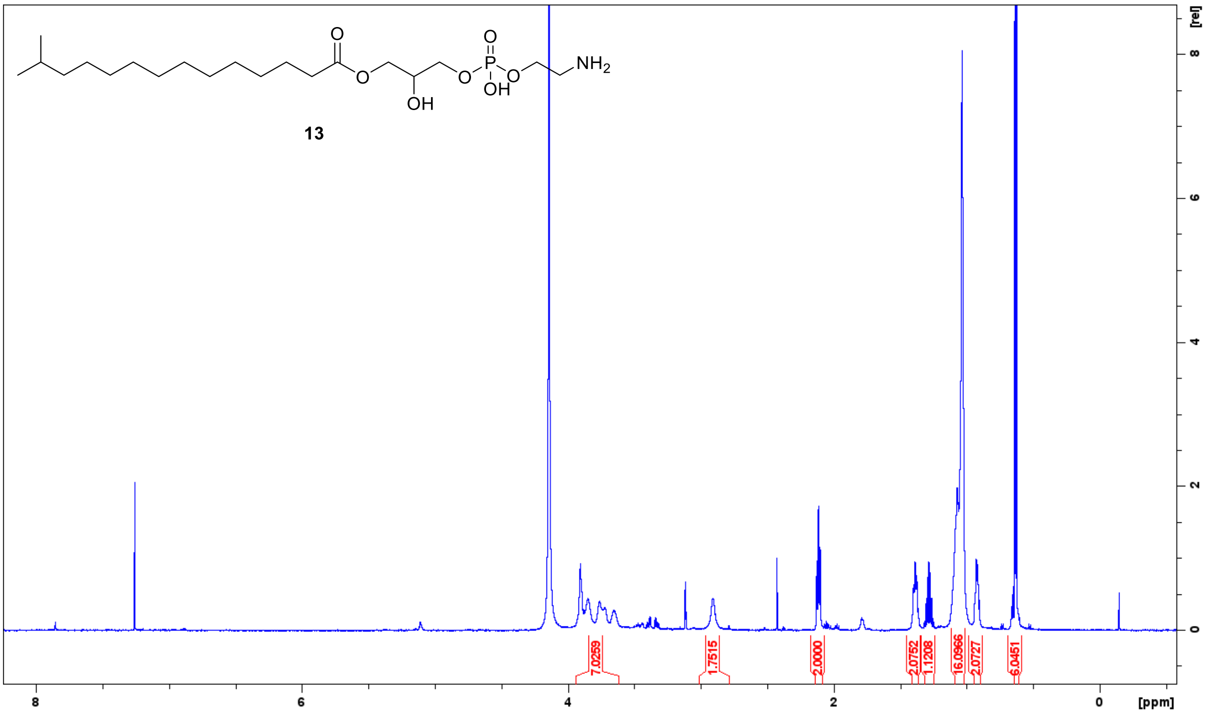

Figure S25. ^{1}H-^{1}H COSY (600 MHz, $CDCl_3$/MeOD-d_4 2:1) of LPE **13**.

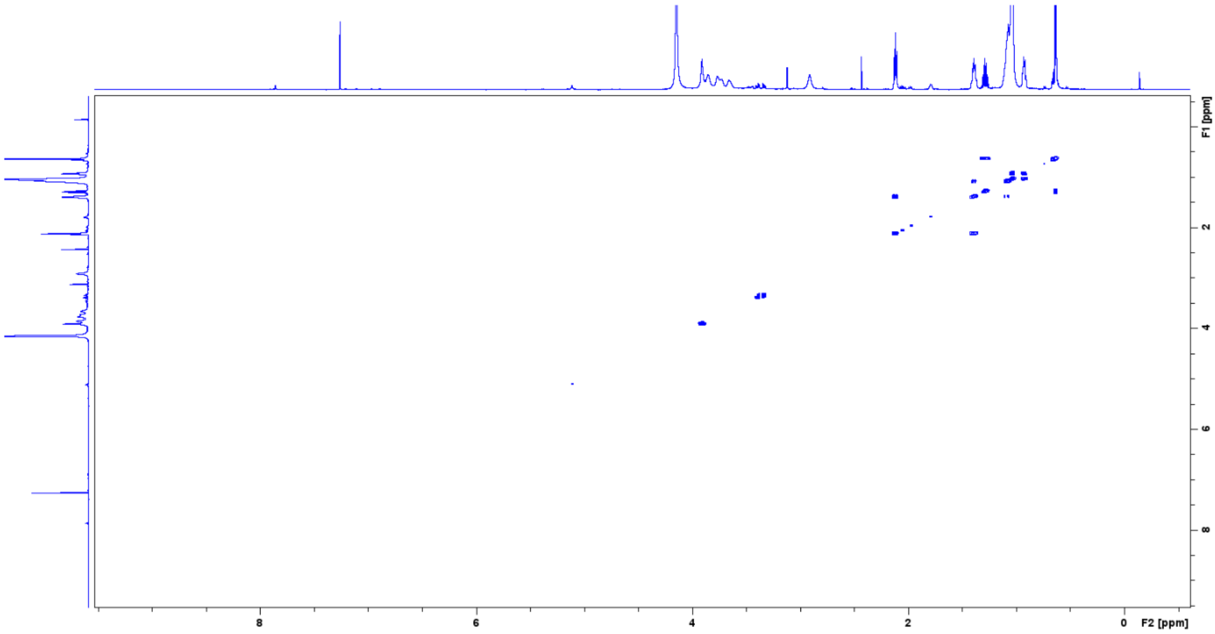

Figure S26. ^{1}H-^{13}C HSQC (600 MHz, $CDCl_3$/MeOD-d_4 2:1) of LPE **13**.

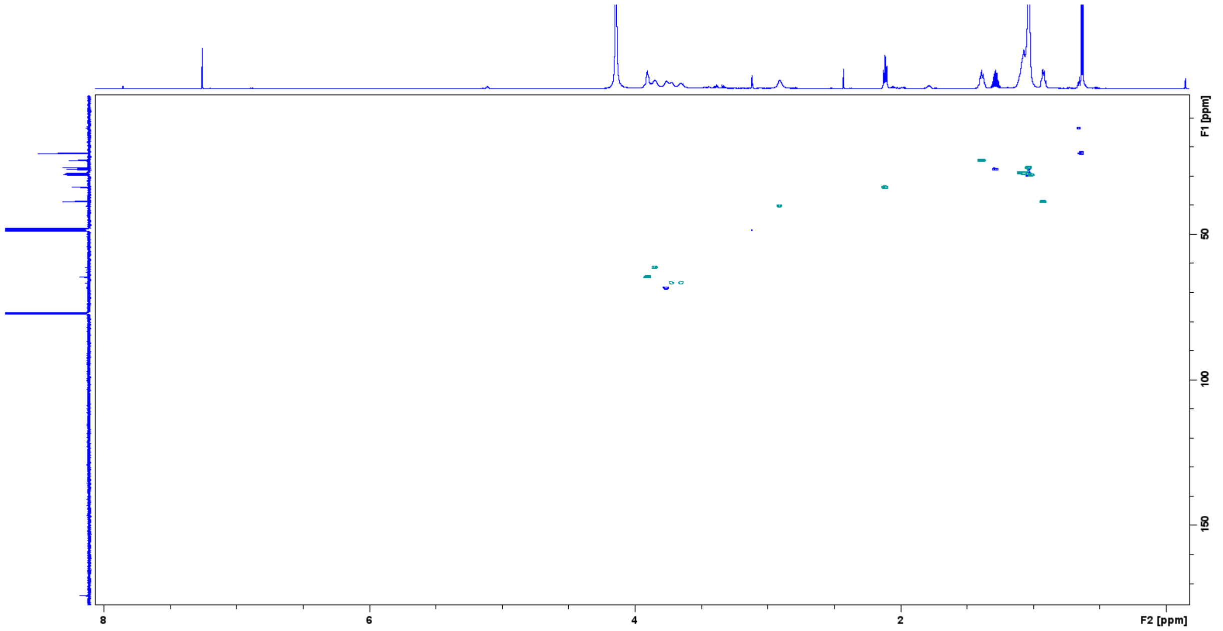

Figure S27. ^{1}H-^{13}C HMBC (600 MHz, $CDCl_3$/MeOD-d_4 2:1) of LPE **13**.

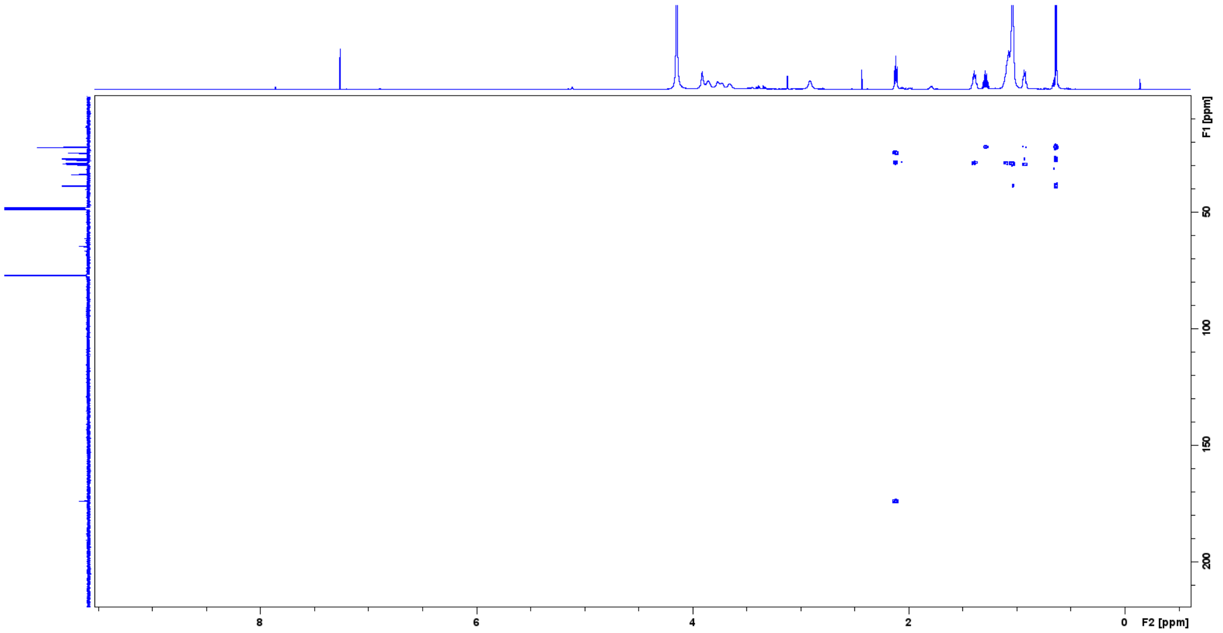

Figure S28. ^{13}C-NMR (151 MHz, $CDCl_3$/MeOD-d_4 2:1) of LPE **13**.

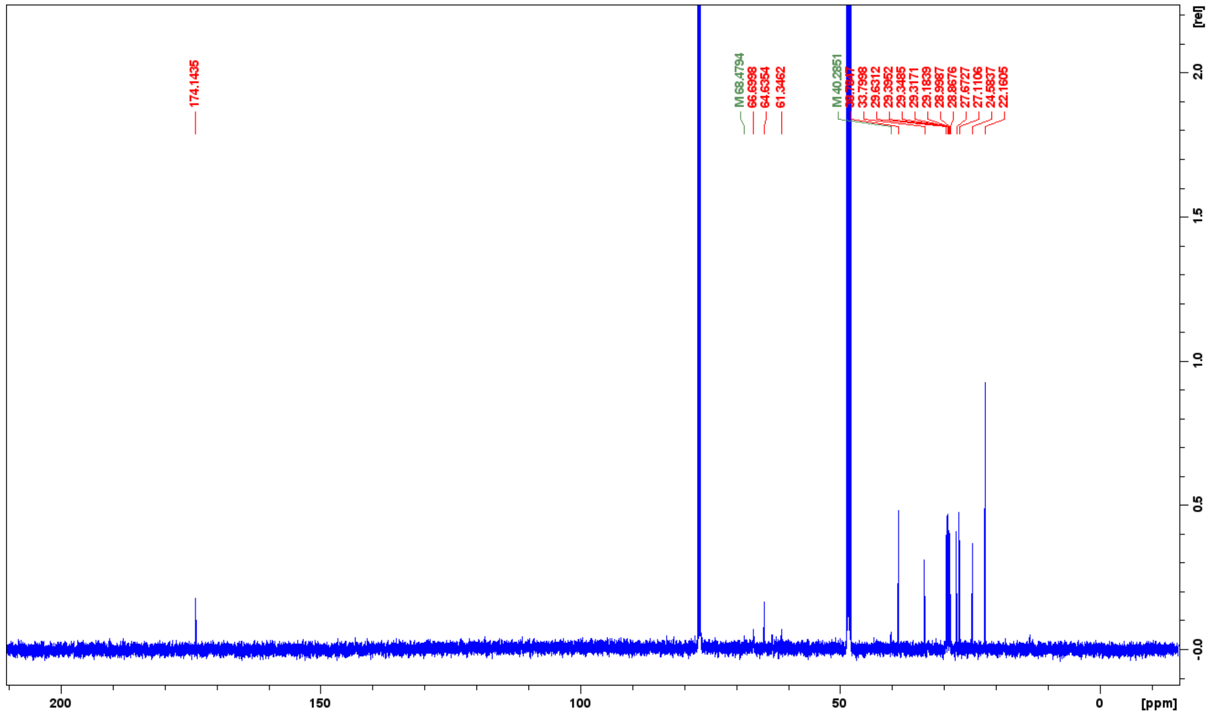

Figure S29. ^{31}P-NMR (243 MHz, $CDCl_3$/MeOD-d_4 2:1) of LPE **13**.

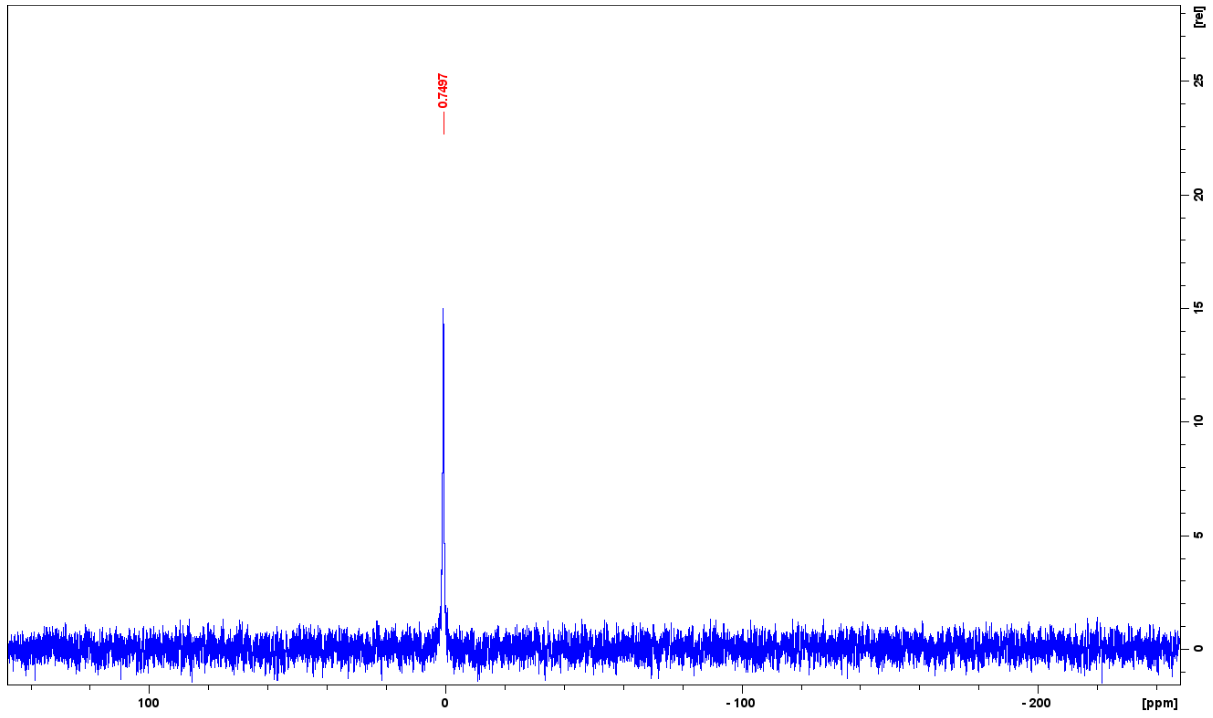

Figure S30. ^{1}H-NMR (400 MHz, MeOD-*d*4) of NAAA **14**.

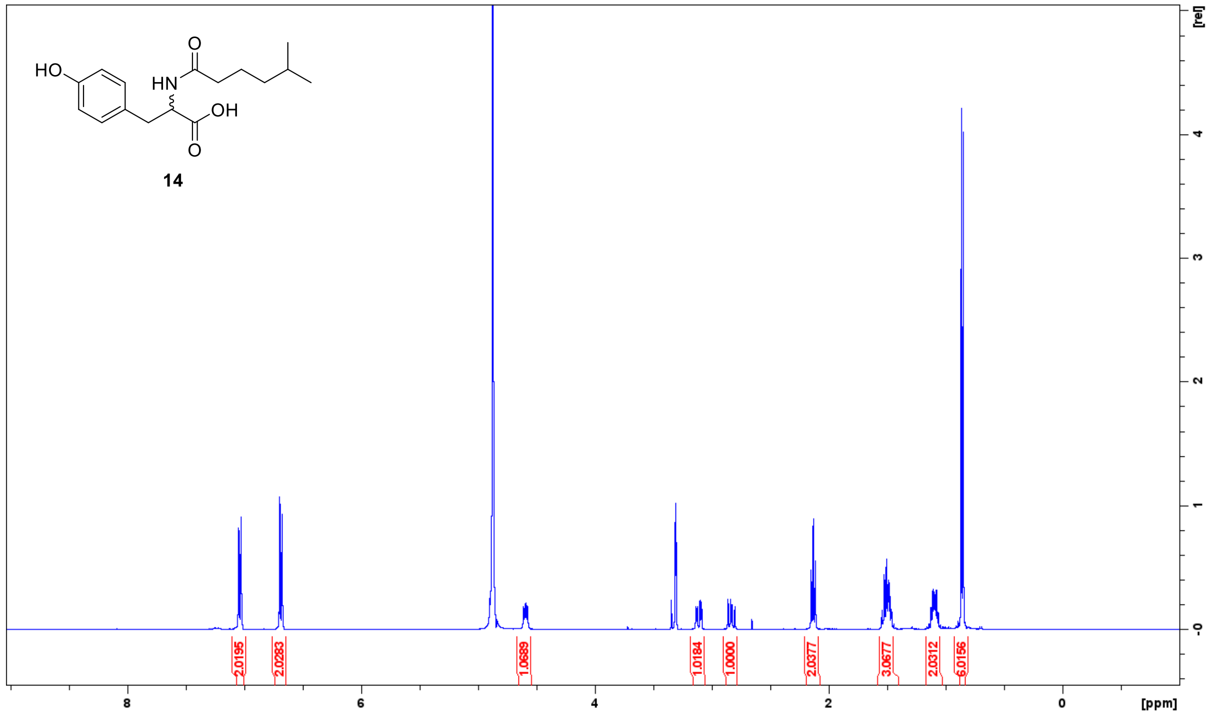

Figure S31. ^{1}H-^{1}H COSY (400 MHz, MeOD-*d*4) of NAAA **14**.

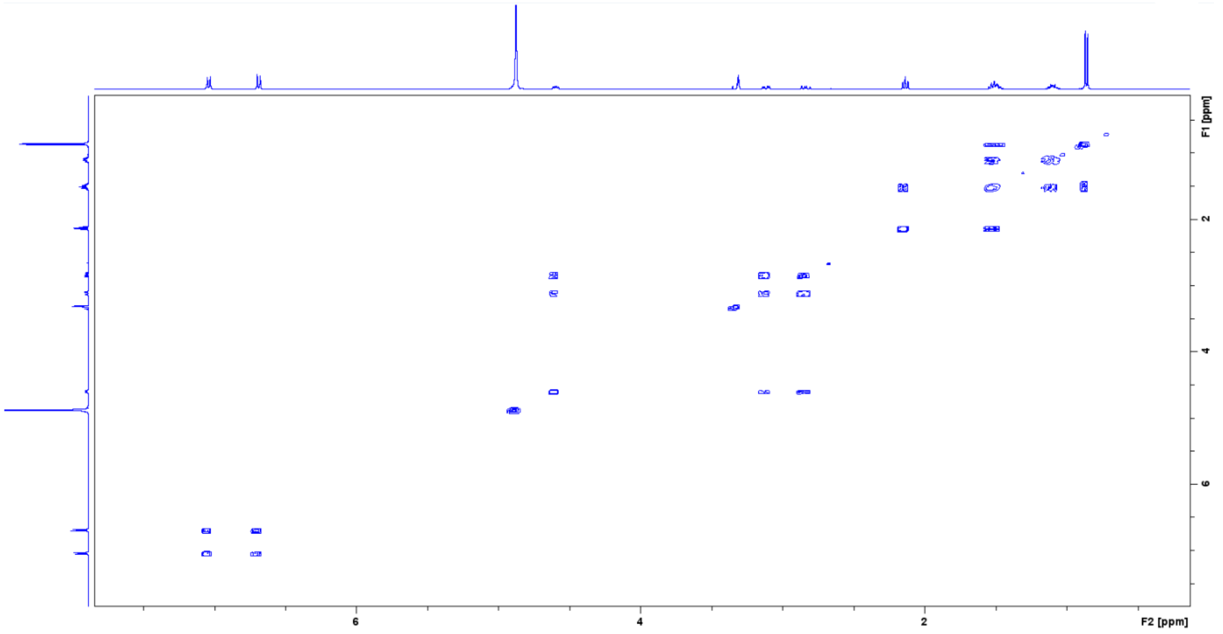

Figure S32. ^{1}H-^{13}C HSQC (400 MHz, MeOD-d_4) of NAAA **14**.

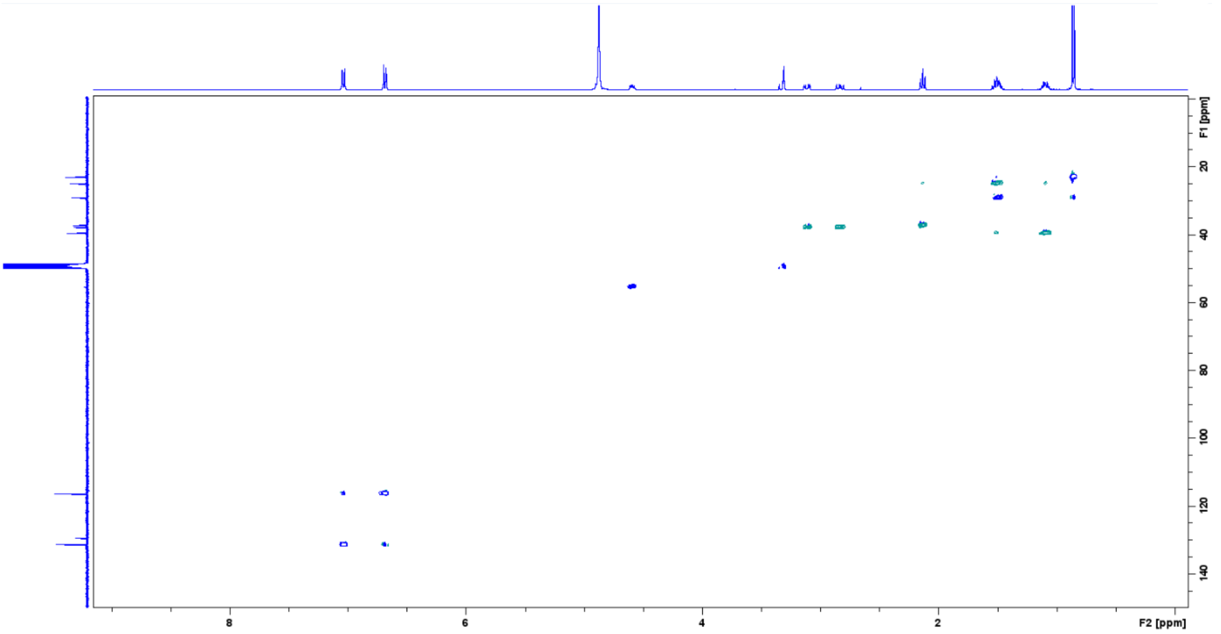

Figure S33. ^{1}H-^{13}C HMBC (400 MHz, MeOD-d_4) of NAAA **14**.

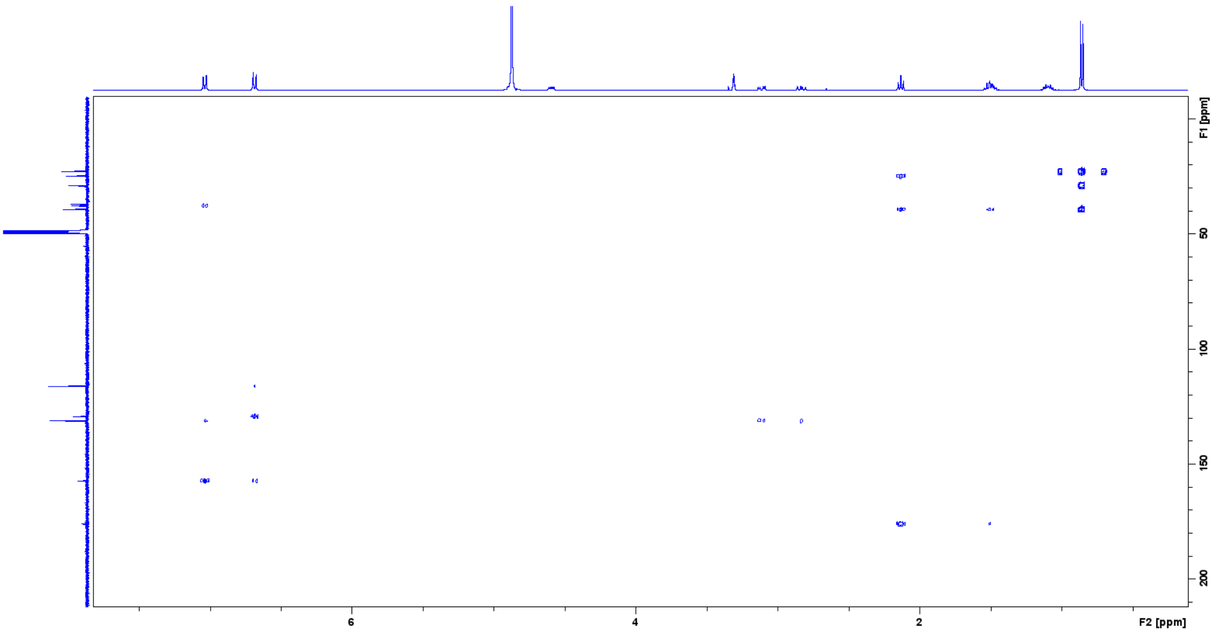

Figure S34. ^{13}C-NMR (101 MHz, MeOD-d_4) of NAAA **14**.

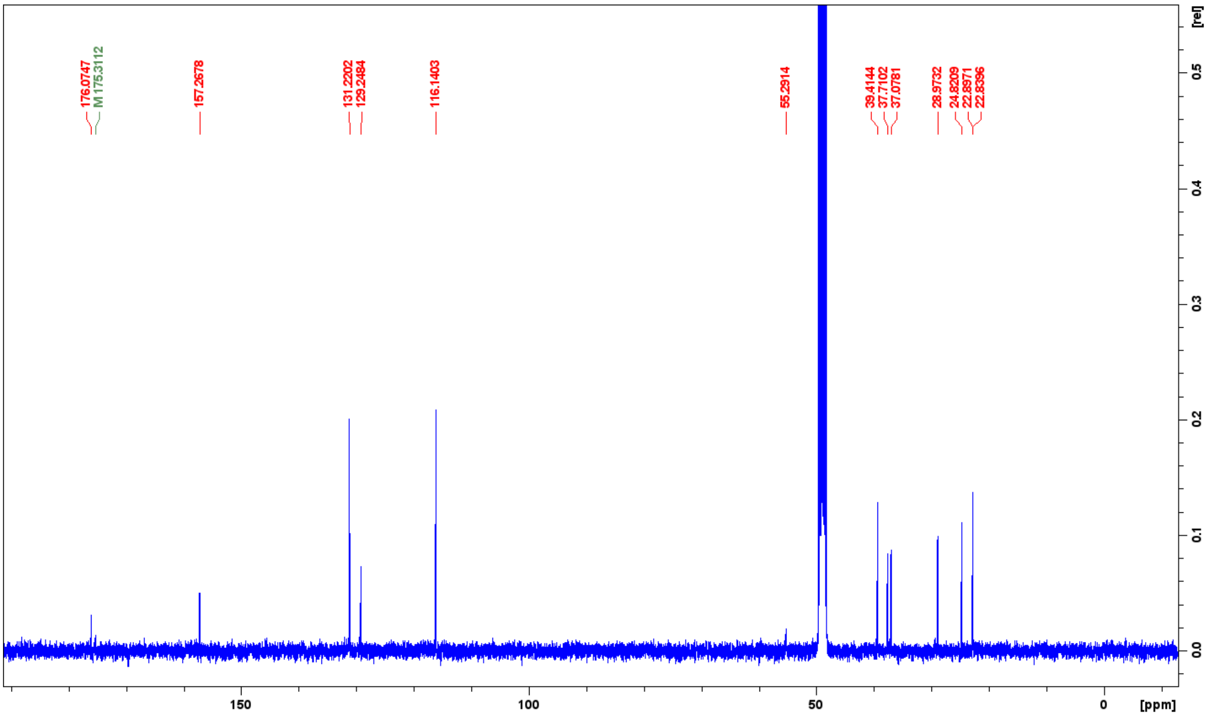

Figure S35. ^{1}H-NMR (400 MHz, MeOD-d_4) of NAAA **15**.

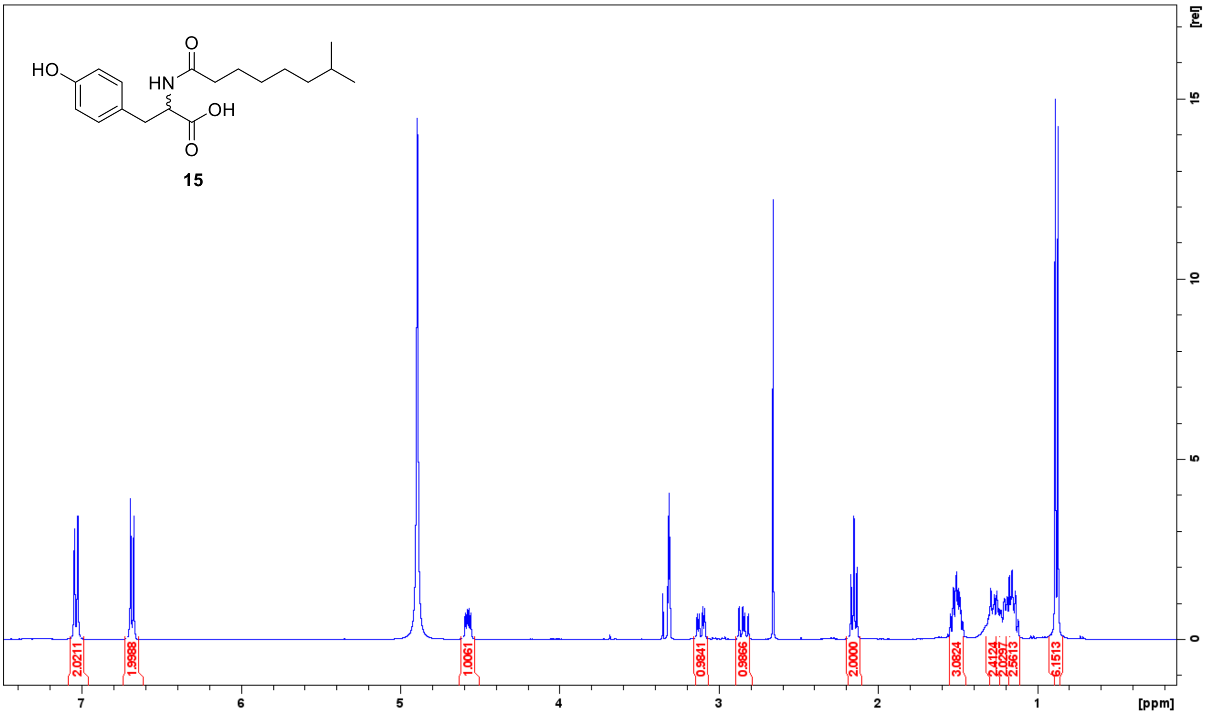

Figure S36. ^{1}H-^{1}H COSY (400 MHz, MeOD-d_4) of NAAA **15**.

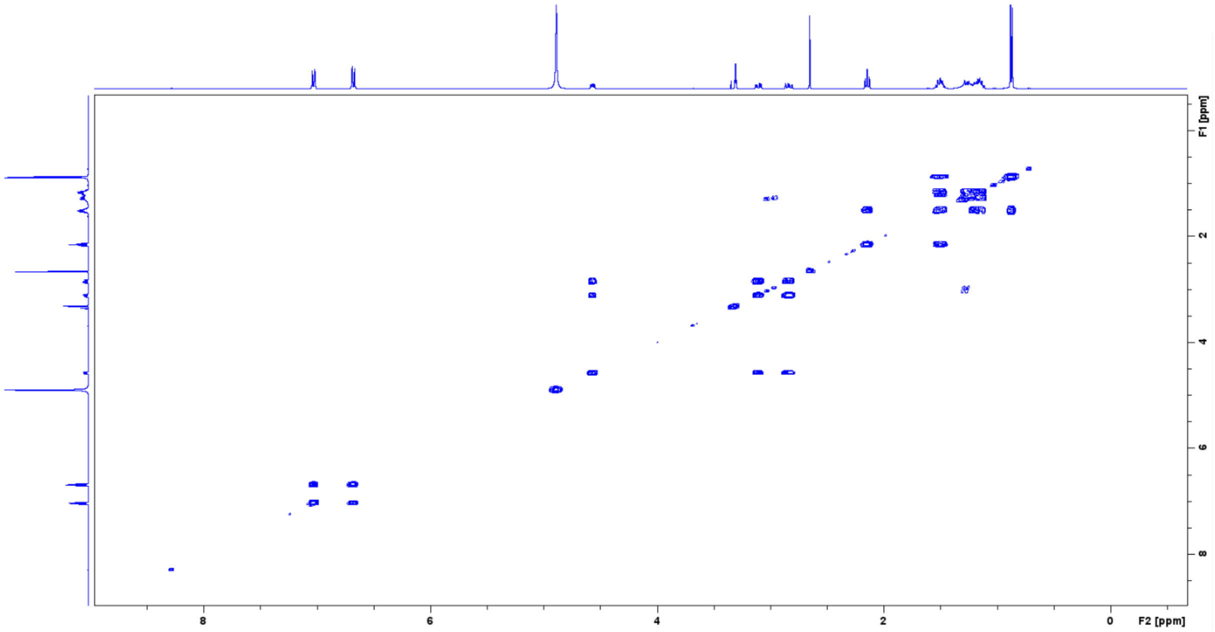

Figure S37. ^{1}H-^{13}C HSQC (400 MHz, MeOD-d_4) of NAAA **15**.

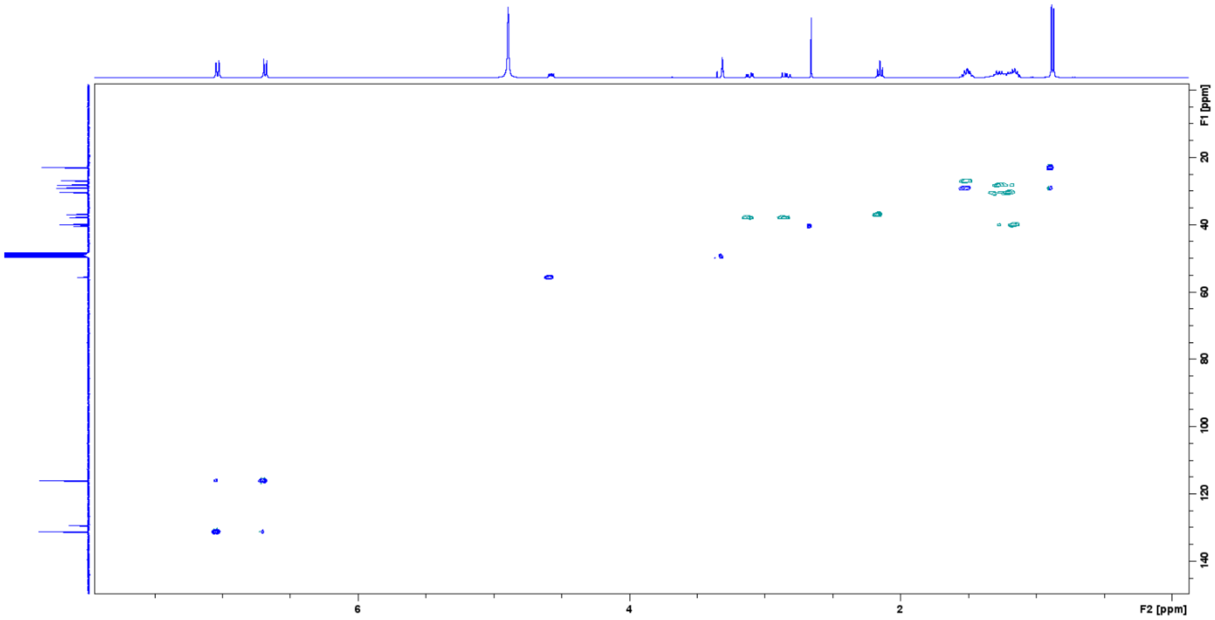

Figure S38. ¹H-¹³C HMBC (400 MHz, MeOD-*d*4) of NAAA **15**.

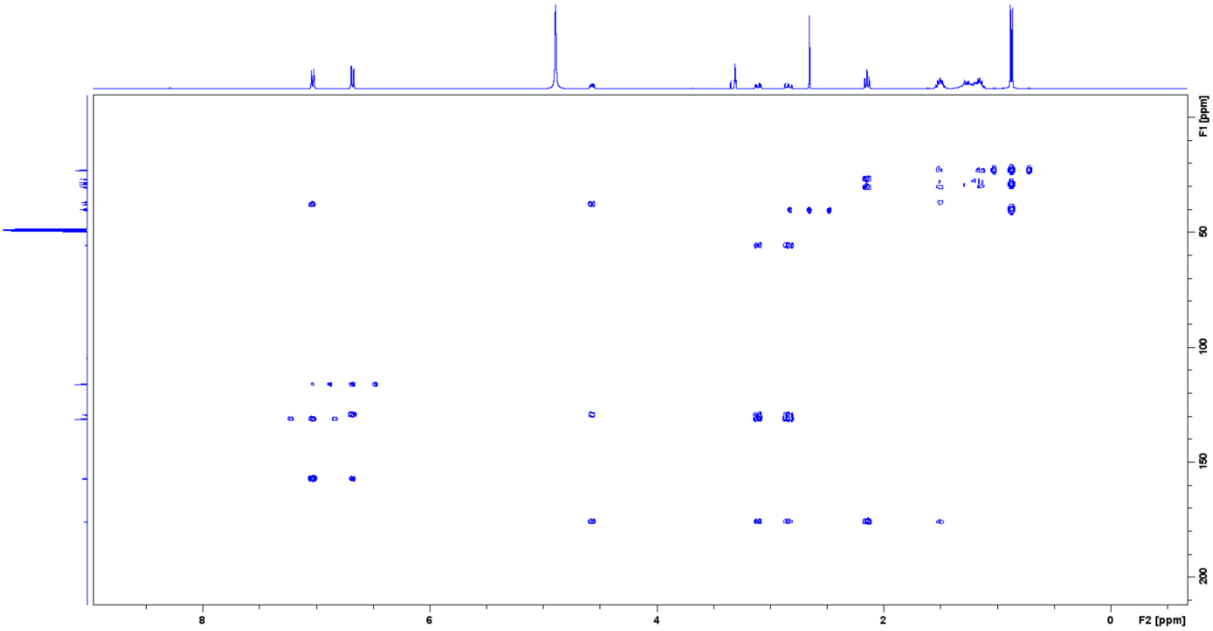

Figure S39. ¹³C-NMR (101 MHz, MeOD-*d*4) of NAAA **15**.

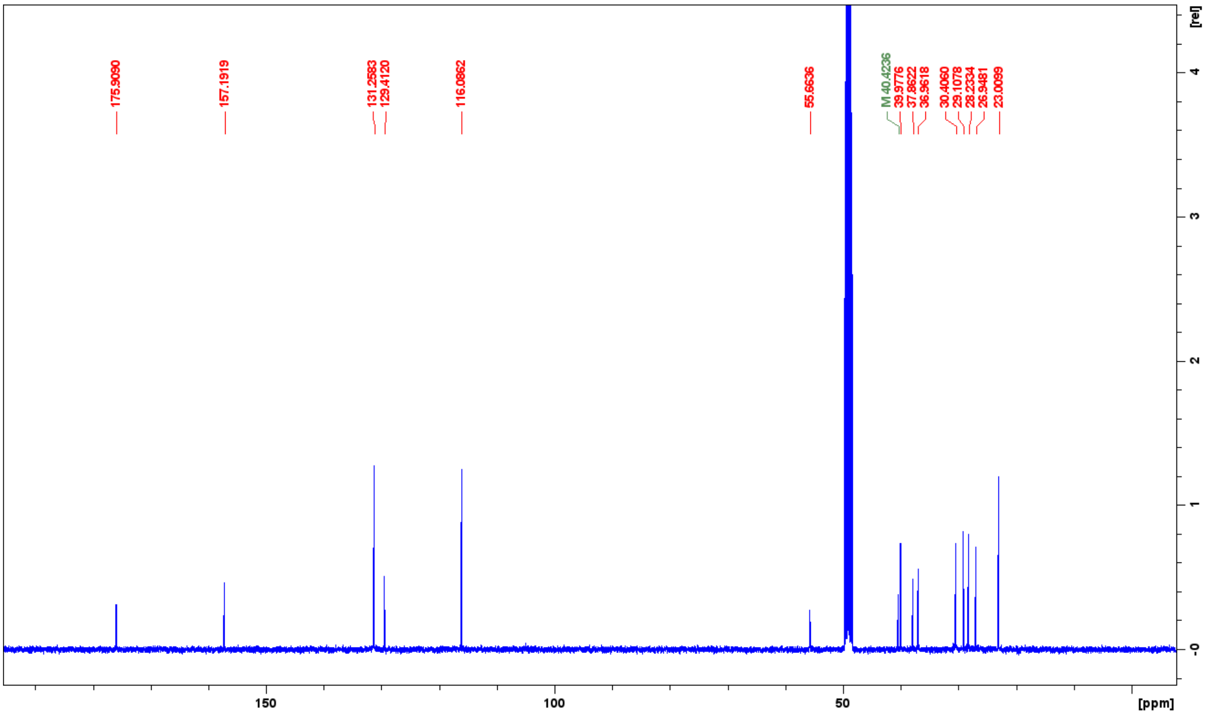

Figure S40. ^{1}H-NMR (400 MHz, MeOD-d_4) of NAAA **16**.

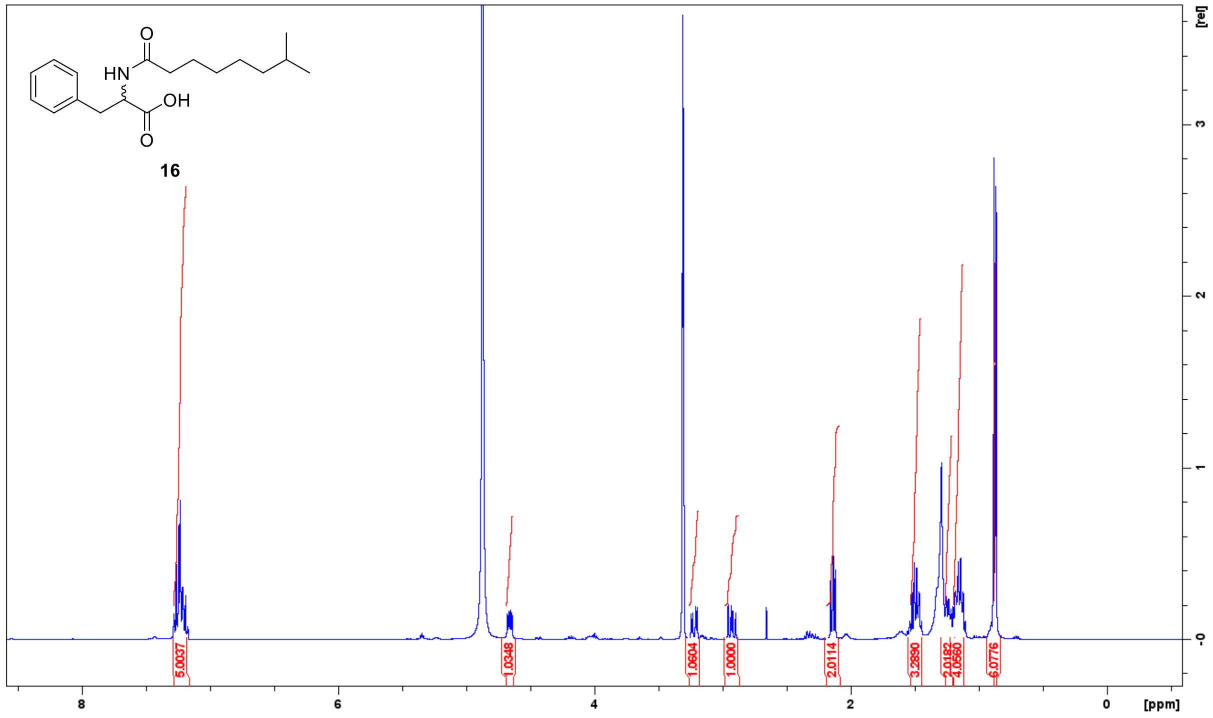

Figure S41. ^{1}H-^{1}H COSY (400 MHz, MeOD-d_4) of NAAA **16**.

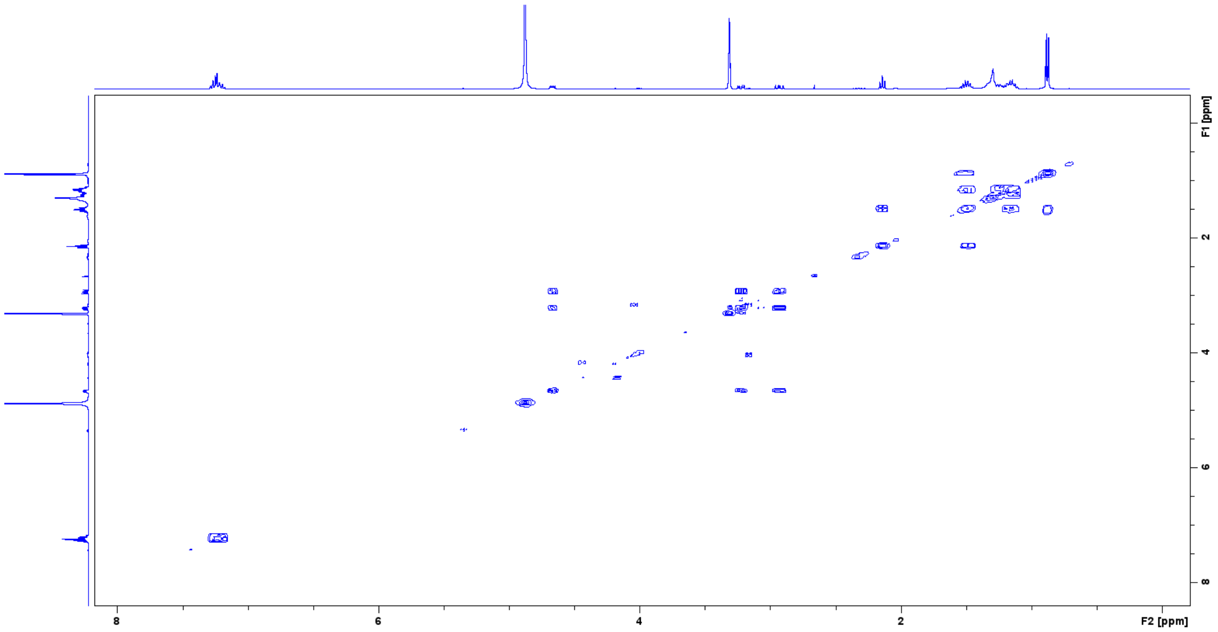

Figure S42. ^{1}H-^{13}C HSQC (400 MHz, MeOD-*d*$_4$) of NAAA **16**.

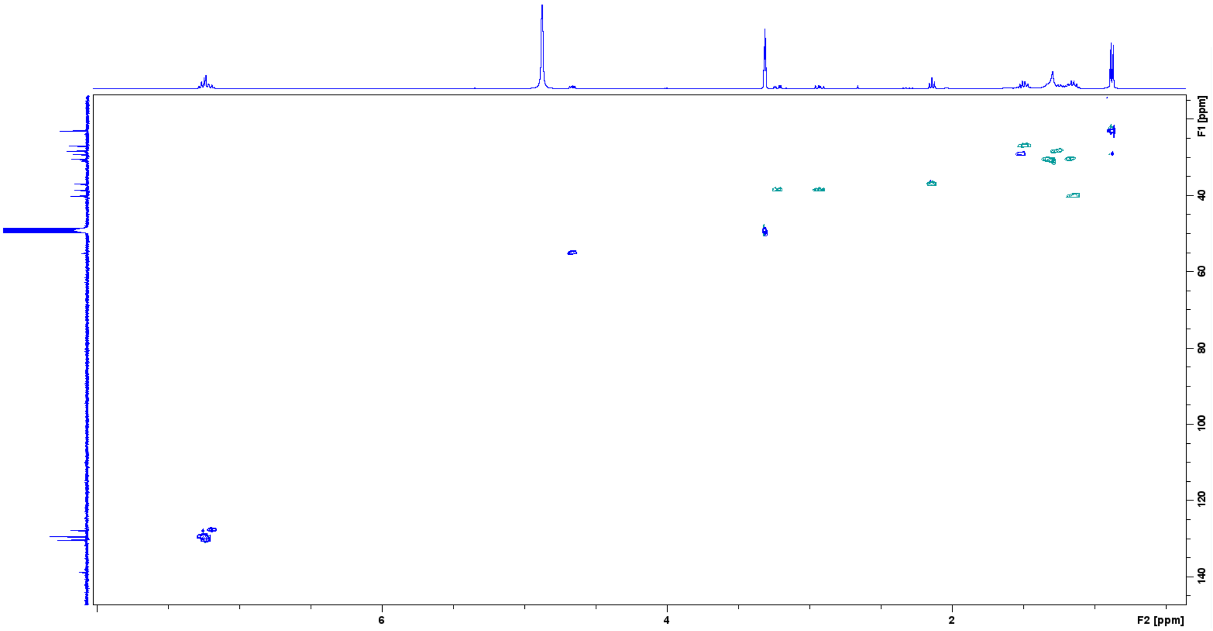

Figure S43. ^{1}H-^{13}C HMBC (400 MHz, MeOD-*d*$_4$) of NAAA **16**.

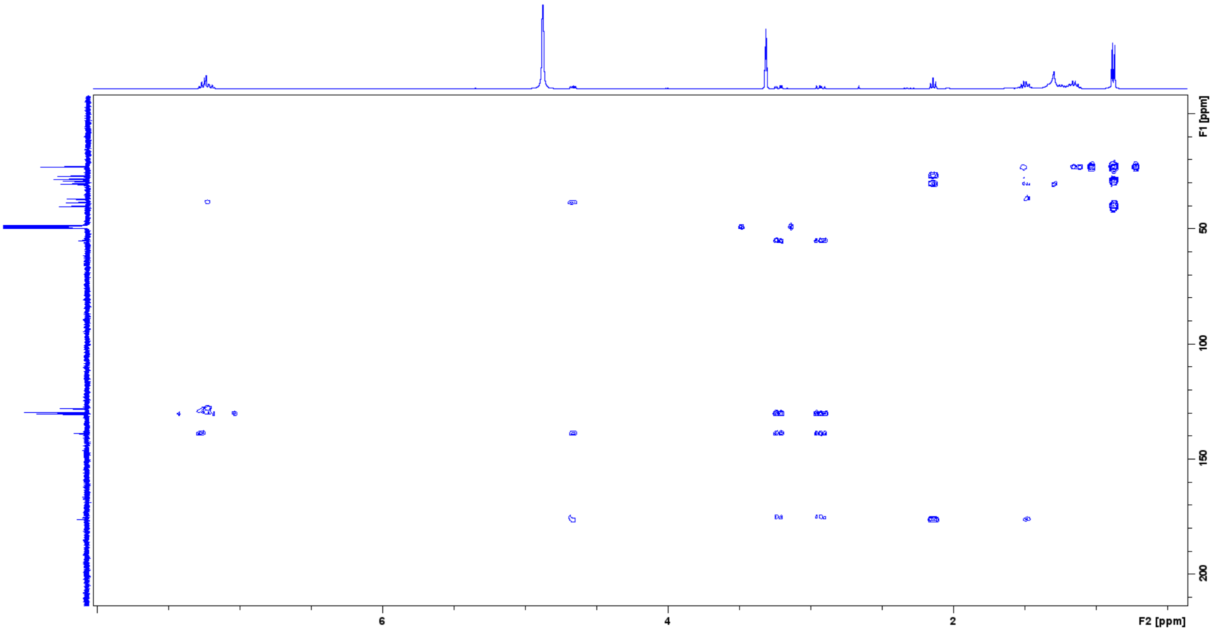

Figure S44. ^{13}C-NMR (101 MHz, MeOD-d_4) of NAAA **16**.

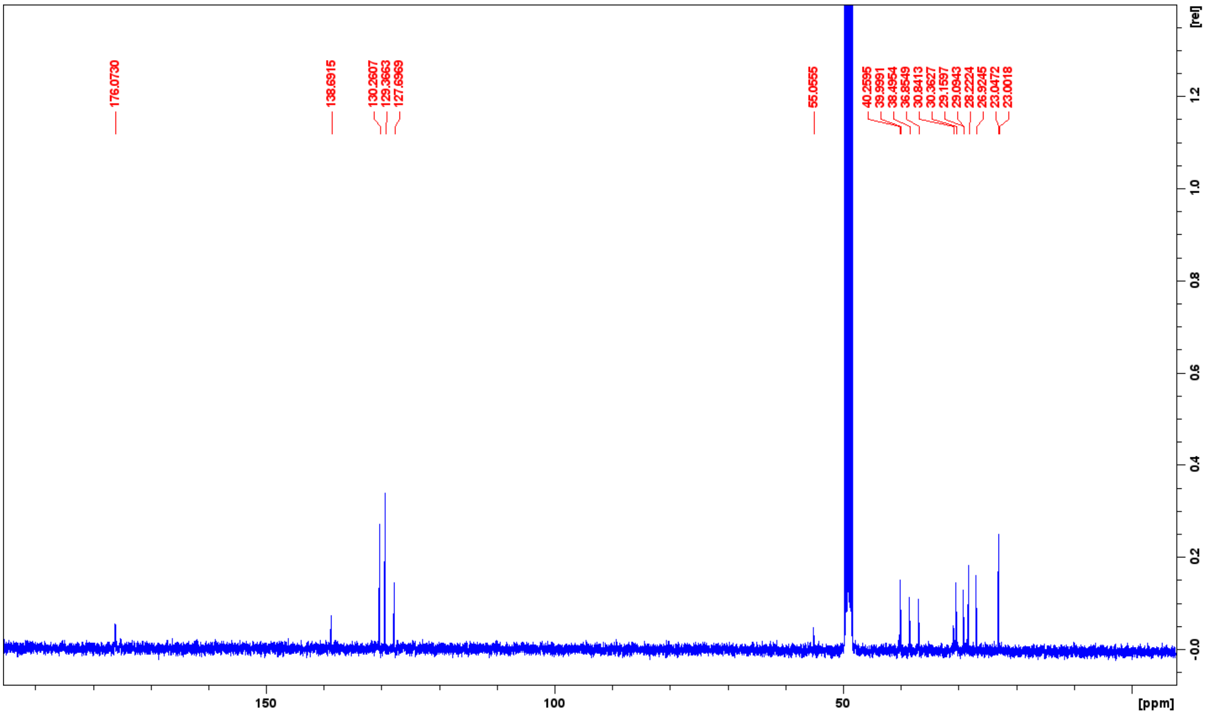

Supplement: Supplementary file 1 [file molecules-26-05195-s001.zip › molecules-1343842-supplementary/molecules-1343842-supplementary.pdf]
